# A novel batch-effect correction method for scRNA-seq data based on Adversarial Information Factorization

**DOI:** 10.1371/journal.pcbi.1011880

**Published:** 2024-02-22

**Authors:** Lily Monnier, Paul-Henry Cournède

**Affiliations:** 1 Paris-Saclay University, CentraleSupélec, Laboratory of Mathematics and Computer Science (MICS), Gif-sur-Yvette, France; University of Wisconsin, Madison, UNITED STATES

## Abstract

Single-cell RNA sequencing (scRNA-seq) technology produces an unprecedented resolution at the level of a unique cell, raising great hopes in medicine. Nevertheless, scRNA-seq data suffer from high variations due to the experimental conditions, called batch effects, preventing any aggregated downstream analysis. Adversarial Information Factorization provides a robust batch-effect correction method that does not rely on prior knowledge of the cell types nor a specific normalization strategy while being adapted to any downstream analysis task. It compares to and even outperforms state-of-the-art methods in several scenarios: low signal-to-noise ratio, batch-specific cell types with few cells, and a multi-batches dataset with imbalanced batches and batch-specific cell types. Moreover, it best preserves the relative gene expression between cell types, yielding superior differential expression analysis results. Finally, in a more complex setting of a Leukemia cohort, our method preserved most of the underlying biological information for each patient while aligning the batches, improving the clustering metrics in the aggregated dataset.

## Author summary

Single-cell RNA sequencing captures the signal of individual cells, allowing a finer resolution than bulk sequencing, which is particularly important for studies comprising rare populations like tumor heterogeneity or lineage tracing studies. However, it is sensitive to the experimental conditions, which induce a bias in the data, called batch effects. Those technical variations hinder any aggregated analysis, limiting scRNA-seq to individual trials. To address this issue, we developed a novel Deep-Learning method called Adversarial Information Factorization, which aims at factorizing the batch effects from the biological signal to align the individual trials for downstream aggregated analysis. The model is trained to learn the batch-conditional cells’ distributions and then corrects batch effects by projecting all cells onto the same batch distribution.

This is a *PLOS Computational Biology* Methods paper.

## Introduction

**Fig 1:**
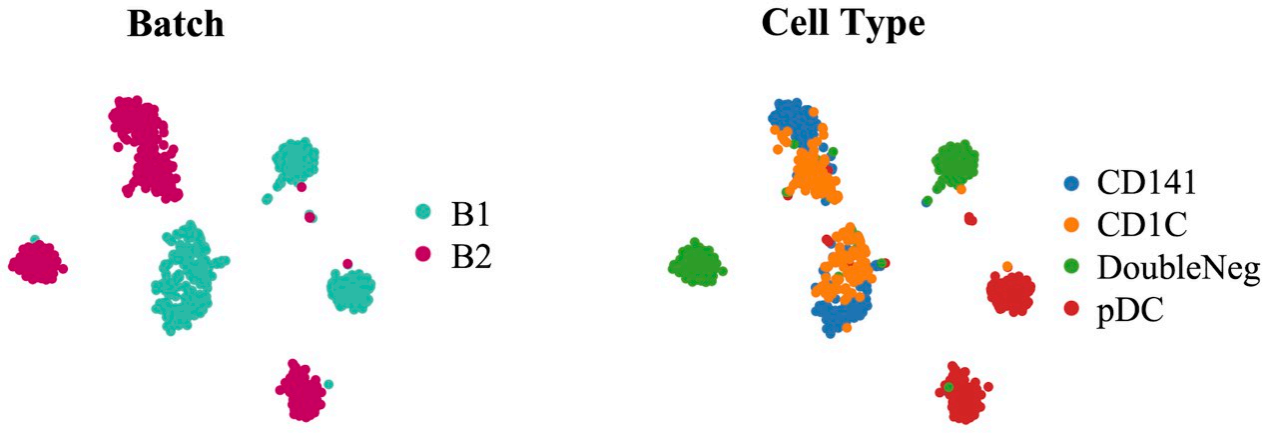
t-SNE visualization of a scRNA-seq dataset of human dendritic cells. This dataset [10] is composed of 4 blood cell types and 2 batches, colored by either batch label (left) or cell type label (right).

Single-cell RNA sequencing was developed to characterize high-throughput gene expression profiles for populations of individual cells. The main benefit of scRNA-seq, compared to bulk-sequencing, is its ability to capture an unprecedented resolution of cellular heterogeneity in complex tissues [1], thus allowing the study of cell-specific changes in the transcriptome, e.g. cell type identification [2], stochasticity of gene expression [3] and heterogeneity of cell responses [4], or the inference of gene regulatory networks across cells [5]. This technology is particularly convenient for identifying rare cell populations [6] that would have been undetected in a pooled-cells analysis, such as malignant tumor cells within a tumor mass. scRNA-seq is also increasingly employed to trace lineage and developmental relationships between heterogeneous, yet related, cellular states in scenarios such as blood cell differentiation [7] or cancer mutational evolution [8]. Thus, it is becoming widely used across biological disciplines, including neurology [9], oncology, and immunology. However, for comparison or aggregation purposes, it is usually necessary to integrate scRNA-seq datasets from different origins. scRNA-seq experiments are often prone to technical variations due to differences in handling the trials, possibly at different times, by different experimenters, in different tissues, or with different technologies. This bias, induced by each individual trial, is called batch effect and can confound biological variations of interest during data integration. Hence, they may hamper downstream analyses and make results inconclusive. Fig 1 illustrates this phenomenon with a t-SNE visualization of a study presented by [10] that gathered scRNA-seq expression of human dendritic cells and annotated their types. This subset comprises four cell types (pDC, CD1c+, CD141+, and Double Negative) and two batches. Each shared cell type is split with respect to the batch label, showing how the batch effects could deteriorate a clustering task.

Many methods were designed to correct RNA-seq batch effects, focusing first on bulk sequencing and later on scRNA-seq data as the new technology emerged. A wide variety of algorithms exists, based on different theoretical backgrounds: from a Bayesian approach (ComBat [11], Limma [12], BUS [13]) to Correlation analysis (Seurat [14–16]) through Nearest-neighbors (MNNCorrect [17], fastMNN [18], BBKNN [19], BEER [20], Scanorama [21]), Deep-Learning (MMD Resnet [22], scGen [23], scVI [24], BERMUDA [25], DESC [26], ResPAN [27]) and others (fRMA [28], SCAN [29], SVA [30], LIGER [31], Harmony [32]). Nevertheless, those methods were usually developed to answer a particular use case, limiting their use in real-world applications: correlation analysis wrestles with extremely imbalanced datasets; nearest-neighbors models require at least one shared cell type across batches and do not scale well to large datasets; MNNCorrect does not support multiple batches; BBKNN, Harmony, and LIGER do not allow any other downstream task than clustering, as the first method returns a connectivity graph instead of the corrected data, and the two others rely on a clustering task in an embedded space; scGen requires the prior knowledge of the cell types, etc. As underlined in the review [33], no method is versatile enough to yield state-of-the-art results in all scenarios.

The numerous features (tens of thousands) produced by the scRNA-seq technology and the potentially large number of single cells analyzed make deep learning models strong candidates for capturing the underlying biological signal, as exposed in [34]. Inspired by the ability to reconstruct images conditionally to an attribute (smiling or not smiling) presented in [35], we propose an architecture relying on adversarial information factorization to answer the batch effect correction problem in scRNA-seq data. The rationale behind our idea is to enable the reconstruction of a sample conditionally to a batch label, thus allowing the alignment of the datasets by projecting the cell distributions of different batches onto a single one. To assess the proposed model’s performance against the state-of-the-art, we selected use cases from the benchmark proposed by [33] and improved their evaluation framework. We focused on three major criteria: the ability to deal with a low signal-to-noise ratio, batch-specific cell types, and imbalanced multi-batches and cell types, encompassing most of the current models’ deficiencies.

## Methods

### Adversarial Information Factorization

#### Model presentation

**Fig 2:**
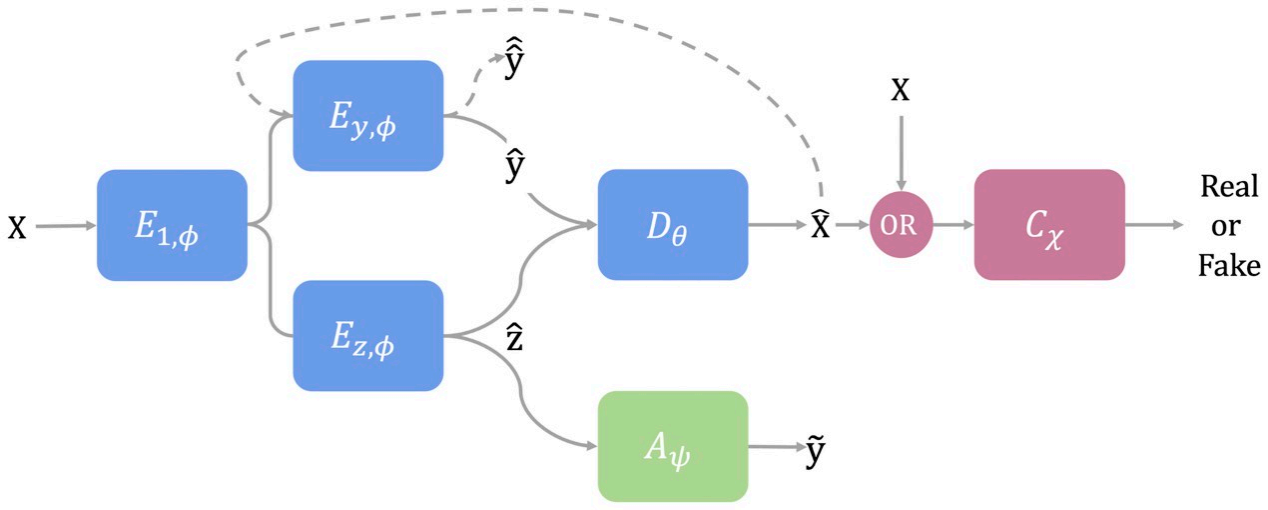
Adversarial Information Factorization’s architecture (AIF). The model comprises three blocks: the CVAE in blue, the GAN network in pink, and the auxiliary network in green. *x* is the original cell’s gene expression, *y* is the true batch label, $\hat{z}$ is the latent vector, $\hat{x}$ is the reconstructed cell’s gene expression, $\hat{y}$, $\hat{\hat{y}}$, $\tilde{y}$ are the predicted batch labels based on *x*, $\hat{x}$ and $\hat{z}$ respectively.

Our method is inspired by a model developed by [35], which enables the conditional generation of celebrities’ faces (e.g., smiling or not). Its backbone relies on a Conditional Variational Auto-Encoder (CVAE) (in blue in Fig 2), which we adapted to learn batch-conditional distributions of cells. Let $x\in R^{d}$ be the original cell’s gene expression and *y* its batch label. The encoder *E*_*ϕ*_ aims to deconvolve the biological signal from the batch effects in the original cell’s gene expression *x*, by encoding the batch information in $\hat{y}$ and the biological information in a latent vector $\hat{z}\in R^{k}$ sampled from the shared latent space, i.e., supposedly deprived of batch effects. The latent space follows a multivariate Gaussian $N(\mu_{q_{\phi }},\Sigma_{q_{\phi }})$, whose parameters are inferred by the encoder. The decoder *D*_*θ*_ learns how to reconstruct the cells’ distributions conditionally to the batch from the latent vector $\hat{z}$ and the predicted batch label $\hat{y}$. The CVAE is trained on a multi-objective involving:

- a reconstruction loss $L_{rec}$, embedded by the Mean Square Error (MSE) between the original and the reconstructed cells, respectively *x* and $\hat{x}$, which forces the reconstructed cells to be close to the original cells,
- a Kullback-Leibler (*KL*) divergence between the posterior distribution *q*_*ϕ*_ in the latent space and the prior *p*, which provides regularization in the latent space,
- a classification loss $L_{class}$, defined as a cross-entropy ($L_{ce}$) between the true batch label *y* and the predicted batch label $\hat{y}$, ensuring that the model accurately predicts the batch label.

To benefit from both the CVAE’s and Generative Adversarial Network’s (GAN) generative properties, [35] added a discriminator network *C*_*χ*_ (in pink in Fig 2). It is trained to predict if the sample is real (i.e., original) or fake (i.e., reconstructed), using a cross-entropy classification loss $L_{GAN}$, which is also added as an adversarial term to the encoder’s objective to incite the generation of realistic samples. The AIF’s specificity resides in the additional auxiliary network *A*_*ψ*_ (in green in Fig 2), trained to predict the batch label $\tilde{y}$ from the latent representation $\hat{z}$, using a cross-entropy classification loss $L_{aux}$. This term is adversarially incorporated into the encoder’s objective to force the encoder’s representation $\hat{z}$ to fool the batch label classifier. This batch information factorization constraint ensures that the biological signal, embedded in $\hat{z}$, is deprived of any batch effects. In Fig 2, the dashed lines show how reconstructed samples are passed back through the encoder to obtain a predicted batch label $\hat{\hat{y}}$ based on the reconstruction. It is then compared to the true batch label *y* using a cross-entropy loss, denoted ${\hat{L}}_{class}$, which reinforces the model’s ability to retain the batch information in the reconstructed samples. We added a projection constraint $L_{proj}$ to the original AIF model to provide regularization in the latent space and cross-over between the batch-conditional distributions. The decoder now has access to all cell types’ latent distributions for each batch, addressing the batch-specific cell types’ singularity. It proved particularly beneficial for batch-specific cell types or very noisy data and the differential expression analysis as it removed most of the negative gene expression induced by projection (Table C in S4 Appendix). It consists of a cosine similarity ($L_{cos}$) between the reconstructed samples $\hat{x}$ and their projections onto the average batch (i.e. using $\hat{y}=0.5$) and a randomly chosen batch, ${\hat{x}}_{avg}$ and ${\hat{x}}_{rand}$ respectively. The maximization of this constraint during the AIF model’s training ensures the similarity between reconstructed cells from different batches.

In summary, we obtain the following losses to train the encoder and the decoder:

$$\begin{array}{c} \left\{ \begin{array}{cc} L_{enc}= & L_{rec}+\alpha L_{KL}+\rho L_{class}+\beta {\hat{L}}_{class}-\mu L_{proj}-\delta L_{gan}-\gamma L_{aux}\\ L_{dec}= & L_{rec}+\beta {\hat{L}}_{class}-\delta L_{gan}-\mu L_{proj}\; \end{array}\right. \end{array}$$ (1)

with $L_{rec}=MSE\left(\hat{x},x\right)$ $=\sum_{j=1}^{d}{\left(x_{j}-{\hat{x}}_{j}\right)}^{2}$, $L_{KL}=KL[q_{\phi }(z|x)\left|\right|p\left(z\right)]$, $L_{class}=L_{ce}\left(\hat{y},y\right)$, ${\hat{L}}_{class}=L_{ce}\left(\hat{\hat{y}},y\right)$, $L_{aux}=L_{ce}\left(\tilde{y},y\right)$, $L_{proj}=L_{cos}\left(\hat{x},{\hat{x}}_{avg}\right)+L_{cos}\left(\hat{x},{\hat{x}}_{rand}\right)$, $L_{gan}=\frac{1}{3}\left(L_{ce}(y_{real},C_{\chi }(x))+L_{ce}(y_{fake},C_{\chi }(D_{\theta }(E_{\phi }(x))))+L_{ce}(y_{fake},C_{\chi }(D_{\theta }(z)))\right)$.

For two classes, $L_{ce}\left(\hat{y},y\right)=y\text{log}\left(\hat{y}\right)+\left(1-y\right)\text{log}\left(1-\hat{y}\right)$.

Since $q_{\phi }\left(z|x\right)∼N\left(\mu_{q_{\phi }},\Sigma_{q_{\phi }}\right)$ and p(z)∼N(0,1): $L_{KL}=\frac{1}{2}[-\text{log}|\Sigma_{q_{\phi }}|-k+\mu_{q_{\phi }}^{T}\mu_{q_{\phi }}+tr\left(\Sigma_{q_{\phi }}\right)]$.

We designed the CVAE’s structure based on scGen’s VAE architecture, using their encoder’s and decoder’s backbones. We implemented a loss normalization using the losses’ medians, computed on the last 10% of the total number of epochs, to address the different orders of magnitude between the losses, crucial for log-normalized counts. It allows a more focal optimization to deal with wrongly optimized losses in the recent steps efficiently (Fig C in S4 Appendix), which often appears with adversarial and multi-task training [36]. This method will be referred to as *AIF dyn*. We used a weighted cross-entropy to tackle imbalanced batches in Datasets 2 and 5.

Refer to S3 Appendix for details on the AIF model.

#### Batch effect correction

Once the model is trained, the idea is to project all batches’ cells’ distributions onto the reference batch. The original samples are fed to the encoder to retrieve their latent representation’s parameters $\mu_{q_{\phi }}$ and $\Sigma_{q_{\phi }}$. Then, a sample is drawn from the corresponding distributions and is reconstructed conditionally to the batch label of interest. It can be any batch label or a combination of batch labels (e.g. 0.5 to project in a space halfway between batch 1 and batch 2), as long as it is the same for all samples. This study used the label corresponding to the most represented batch. The stochasticity induced by random sampling is addressed by multiple sampling and averaging the reconstructed samples obtained, thus improving the robustness of the batch-effect correction step (Table A in S4 Appendix). Doing so, one fully benefits from the distribution aspect of the VAE compared to a deterministic approach in which the estimated $\mu_{q_{\phi }}$ would serve as the latent vector.

### Related works

In [33], an extensive comparison of batch-effect correction methods for scRNAseq data is performed on ten different datasets. We selected four methods that ranked among the best for many test cases while relying on very different theoretical backgrounds: Harmony [32] and scGen [23] yielded top-of-the-shelf results in all scenarios (except batch-specific cell types for the latter), LIGER [31] was particularly efficient in big data and non-identical cell types use cases, Seurat [16] thrived when dealing with batch-specific cell types and multiple batches scenarios. Moreover, Seurat and Harmony have become standard methods for integrating datasets, the former having a significant impact factor and the latter being used to correct batch effects in a database spanning 140 scRNA-seq cancer studies [37]. LIGER and scGen yield great results in the benchmark and rely on theoretical backgrounds similar to AIF’s (Information Factorization for LIGER and VAE for scGen). We included two other Deep Learning methods: scVI [24] and ResPAN [27]. scVI is a widely-used method in the community based on VAE and Information Factorization, resembling the AIF formulation. ResPAN employs an auto-encoder and adversarial training with a discriminator, yielding superior results to scVI.

#### Harmony

Harmony is a method developed by [32] resorting to iterative clustering to align cells from different batches. Beforehand, dimensionality reduction is performed using the Principal Components Analysis (PCA) algorithm. Harmony uses an iterative procedure: 1) group cells into clusters using a variant of soft K-Means clustering, 2) infer each dataset’s correction factor using the difference between the dataset-specific centroids and the global centroids. Nevertheless, Harmony is a clustering-specific batch-effect correction method performed in the PCA subspace, limiting its use to solely clustering downstream tasks and impeding any integration of new samples post-training.

#### LIGER

LIGER, designed by [31], relies on an integrative non-negative matrix factorization (iNMF) method to factor out the batch-specific from the shared biological signal. Then, a shared factor neighborhood graph is built, connecting cells with similar factor-loading neighborhood patterns. Louvain clustering is performed on the resulting graph, and clusters are aligned using quantile normalization on the factor loadings. Nonetheless, LIGER is also a clustering-specific batch-effect correction method performed in an embedded space, restraining its application for any other downstream analysis task and real-world cases, as it does not allow the immediate integration of new samples. Note that before running the iNMF algorithm, LIGER requires its very own pre-processing pipeline: a normalization step, the extraction of highly variable genes, and a features’ scaling step, meaning that it is impossible to train the model on the raw counts or along with a custom pre-processing pipeline.

#### Seurat

Seurat 3, designed by [16], is an improved version of Seurat 2 [15], hinging on Canonical Correlation Analysis (CCA) to perform dimensionality reduction. Seurat 3 identifies anchors, i.e., similar cell states across batches, using Mutual Nearest neighbors (MNN) in the embedded space. Those anchors are refined using cell type similarity based on the shared nearest neighbor graphs. Finally, a batch-effect correction vector is inferred using the cells’ expression profiles. A tree hierarchy based on the batches’ similarities guides the batch integration order for multi-batch datasets. However, this method does not scale well to large datasets due to memory issues raised by the CCA and MNN computations. Moreover, it does not allow the immediate integration of new samples.

#### scGen

scGen is a Deep-Learning-based method developed by [23] combining a VAE and latent space vector arithmetic to model and predict single-cell expression data. In the case of batch-effect correction, scGen trains the network on the aggregated dataset and then removes the batch effects using latent space arithmetic: the cells are re-normalized by batch and cell types. The comparison is also motivated by scGen relying on a simpler version of VAEs, where conditional reconstruction is avoided using latent space arithmetic to realign the datasets. However, it requires prior knowledge of the cell types, which is often unavailable, and places this method in the supervised category.

#### scVI

scVI [24] couples Bayesian modeling with Deep-Learning to model the cells’ distributions. It employs Neural Networks to infer the latent space’s and library size’s multivariate Gaussian distribution parameters. Then, the genes’ expected frequency and dropout are predicted from those variables and the batch label using Neural Networks. Finally, they are combined to obtain the expected counts. Although the authors claim those final counts are batch-effect corrected, they are generated using different batch labels, which would reconstruct the batch effects. Only the latent space can be considered batch-effect corrected, averting any downstream analysis other than clustering. Besides, no specific loss ensures the latent space is deprived of batch effects. We investigated the authors’ recommended architecture (scVI (r)) and an increased complexity one, similar to scGen’s and AIF’s (scVI (i)).

#### ResPAN

ResPAN [27] is a Deep Learning method, relying on a WGAN (Wasserstein GAN) and random walk mutual nearest neighbor pairing to map cells onto a reference dataset. First, the inter-batch pairs are computed on the PCA subspace using Random Walks. Then, an auto-encoder is trained to map one batch onto the reference batch jointly with a discriminator, predicting whether the cells belong to the same batch. The adversarial discriminator’s loss in the auto-encoder’s objective enhances the mapping’s quality by forcing reconstructed cells to be indiscernible from the reference batch’s cells. Note that for k batches, this process must be repeated *k* − 1 times successively. Before running ResPAN, the authors extracted the first 2,000 highly variable genes (HVGs) in either batches. We performed this step on the log-normalized counts for both versions of the datasets since it did not work on the raw counts.

### Evaluation procedure

To assess the model’s performance in correcting batch effects, two main criteria need to be considered:

- The ability to integrate batches, i.e. batch-mixing.
- The ability to retain the underlying biological signal. For the use cases chosen, it will be assessed through the ability to maintain cell-type purity, i.e., keeping cells within a cell type close to each other.

For the simulation, Differential Expression (DE) analysis will be performed after clustering to provide a more thorough evaluation of the biological signal preserved.

#### Clustering metrics

For both the batch mixing and the cell type purity, we used three clustering-related metrics:

- the Adjusted Rand Index (ARI), which measures the percentage of matches between two labeled lists, corrected for chance.
- the Average Silhouette Width (ASW), which evaluates how similar an object is to its own cluster (cohesion) compared to other clusters (separation).
- the Local Inverse Simpson Index (LISI), which relies on the Inverse Simpson Index computed on a fixed-perplexity neighborhood. It represents labels’ diversity within the neighborhood.

Refer to S2 Appendix for detailed definitions.

#### Clustering algorithms

First, a dimension reduction step is performed using t-SNE for the small datasets and UMAP for the human pancreas dataset, which will then serve as support for the metrics computation. Indeed, we deviated from the pipeline proposed by [33] as the clustering on the PCA subspace often failed to capture the biological differences between cell types compared to t-SNE and UMAP, thus wrongfully deteriorating any clustering metrics (Fig C and Table A in S5 Appendix). t-SNE yields better-structured clusters (more compact and well-separated) on the small datasets, while UMAP better captures the difference between cell types for the large dataset thanks to the higher number of components produced. For the ARI, we used K-Means for the small datasets (Datasets 0 and 1) and Louvain for Dataset 2 due to K-Means’ failures (Fig F in S5 Appendix). To prevent penalizing the models solely on a lousy clustering performance, we calculated the metrics on the full datasets with 20 different random seeds and reported their maximum, thus providing a fairer comparison of the models’ intrinsic performance. To account for robustness, we also computed the metrics on randomly sampled 80% of the embedded data and repeated the operation 20 times. For the AML dataset, we performed Louvain clustering on the t-SNE embeddings, since it yielded the best results for both the original and corrected data.

#### Differential Expression

DE analysis aims to detect Differentially Expressed Genes (DEGs) between two groups. It provides a finer assessment of the biological signal’s preservation than clustering-based metrics, as it compares each gene’s relative expression to the ground truth. Following [33], we conducted a likelihood-ratio test [38] between the populations detected by clustering, restricted to the Highly Variable Genes (HVGs) or not, on the simulation datasets as the ground truth can be accessed through the simulations’ design parameters. We performed DE analysis on the log-normalized and the raw batch-effect-corrected counts, as they capture different gene settings. On log-normalized counts, which is the common practice, DE analysis focuses on the genes’ ratio of total gene expression. In contrast, on raw counts, it compares the cell types’ intrinsic genes’ distribution, which can add some relevant insights if the cell types’ total gene expressions are similar enough. The test being highly sensitive to outliers for the raw counts (Table B in S2 Appendix), we clipped each gene’s distribution per cell type to its 0.02 and 0.98 quantiles for this version. We filtered the DEGs called using a log-fold-change threshold, as recommended by [16] since the test’s poor sensitivity usually yields noisy DEGs. To compare the models, we computed an F1 score between the true, i.e., as intended per design, and detected up and down-regulated DEGs to account for precision and recall. We calculated these metrics across a wide range of log-fold-change thresholds to assess the models’ ability to reconstruct DEGs at different levels of expression ratios. Then, the results are integrated using the Area Under the Curve (AUC) of the F1 score’s evolution.

## Datasets

For the clustering task, we selected three datasets spanning very complex use cases: Dataset 0 suffers from high batch effects relative to the biological signal (i.e., low signal-to-noise ratio), Dataset 1 contains imbalanced and very similar batch-specific cell types, Dataset 2 comprises imbalanced and batch-specific cell types combined with imbalanced multi-batches. Those conditions are often encountered in scRNA-seq due to the diversity of the batch effects’ sources and constitute the most complicated situations from [33] to efficiently disentangle the batch effects from the biological signal. We generated two versions from each dataset (except Dataset 2): the raw counts and the log-normalized counts (normalizing the counts using the median count over the dataset, followed by a log1p transformation), further denoted by *dataset i norm log*. Although this normalization is legitimate in the case of different technologies, it relies on the assumption of a constant total gene expression across cells and conditions, which shrinks the inherent biological differences between cells [39]. For the DE task, we retrieved the raw counts and the true DEGs of the first and second simulations in [33] for differential expression analysis (Dataset 3 and Dataset 4, respectively). We also included a clinical application comprising longitudinal scRNA-seq data of AML-diagnosed patients’ bone marrow biopsies (Dataset 5) [40], priorly log-normalized.

### Dataset 0: Low signal-to-noise ratio

Dataset 0 consists of human blood dendritic cells’ (DCs) scRNA-seq data from [10], generated by the same technology and coming from the same tissue. It is composed of two batches containing four different cell types: i) conventional DCs of types CD1c+, ii) conventional DCs of types CD141+, iii) Double Negative (DN) conventional DCs (CD1c-, CD141-), iv) plasmacytoid DC (pDC). All four types are present in both batches, with 768 cells. The batch effect prevails over the biological difference between CD1c+ and CD141+ cells in the raw counts, placing this dataset in the low signal-to-noise ratio category. The preliminary normalization helped decrease the batch effect. Still, it led to a deterioration of the biological signal for some pDC and DN cells, now clustering with CD1c+ cells (Fig A in S1 Appendix).

### Dataset 1: Batch-specific cell types

Dataset 1 is a subset of Dataset 0, where some CD1c+ and CD141+ cells were removed to make those cell types batch-specific. It comprises two batches containing four different cell types, totaling 576 cells. This characteristic strongly increases the difficulty of batch-effect correction: simply speaking, it is no longer possible to match every cell of one batch to cells in the other batch. Here also, although the normalization step decreased the batch effects, it altered the biological signal for some cells, as a new cluster composed of pDC and DN cells emerged (Fig B in S1 Appendix).

### Dataset 2: Batch-specific cell types with imbalanced multi-batches

Dataset 2 consists of 14,767 human pancreas cells spanning 15 cell types and sequenced by different technologies (inDrops for B1 [41], CelSeq2 for B2 [42], SMART-seq2 for B3 [43], and SMARTer for B4 [44] and B5 [45]) resulting in very high batch effects. The number of samples per batch or cell type is highly variable, ranging from 457 to 8,569 for the batches and 5 to 5,100 for the cell types. Moreover, there is no batch containing all cell types. As the datasets generated by SMARTer and CelSeq2 have enormous values compared to the other technologies (10^3^ times higher), we only investigated the pre-processed version for Dataset 2. The normalization led to a subdivision of most cell types’ clusters even within their own batch: for example, alpha and beta cells are divided into multiple clusters for batch B1, and cell types from the last three batches are now spread out across the embedded space, showing that it modified the batch effects and potentially altered the biological signal (Fig C in S1 Appendix). To assess the model’s ability to generalize to unseen data, we split this dataset into train (80%) and test (20%) (see Table C in S1 Appendix for details).

### Datasets 3 and 4: Simulated datasets with unbalanced cell types and batches

Datasets 3 and 4 are simulated datasets comprising two imbalanced cell types (411 and 989 cells for cell types 1 and 2, respectively) and batches (500 and 900 cells for B1 and B2, respectively), with small and large dropout factors (≈ 0.05 and ≈ 0.25 for Datasets 3 and 4 respectively). From Dataset 3, we generated two other versions by downsampling the first cell type to 101 and 200 cells, further referred to as Dataset 3 (*n*_1_ = 100) and Dataset 3 (*n*_1_ = 200), to investigate the models’ performance when faced with extremely unbalanced cell types and a fewer number of total cells.

### Dataset 5: Effects of chemotherapy on AML-diagnosed patients

This scRNA-seq dataset constitutes a real-world clinical application on the effects of chemotherapy on a cohort of 16 AML-diagnosed patients [40]. The authors sequenced patients’ bone marrow biopsies at different time points: at diagnosis (D0) and after undergoing chemotherapy for most patients (*D*_*i*_, *i* > 0), possibly regrouping multiple time points for a total of 30,334 cells. It encapsulates a much more challenging scenario since the numbers of batches and cell types are important (35 batches and 21 cell types). Both are very unbalanced (from 73 to 3,813 cells per batch and 104 to 6,381 cells per cell type), meaning that the model has to learn many conditional distributions while capturing a fine-grained resolution to preserve the least represented cell type. Besides, the samples contain both malignant and healthy cells, whose mixture drastically changes over time. The cell types span the hematopoiesis’ differentiation tree, resulting in some continuous cell types’ distribution, for example, ProMono-like and Mono-like (Fig D in S1 Appendix). The batch effects reside in both the patient’s specific signal and the sampling time due to technical variations. However, they are intertwined with some true biological variation. Some cell types are patient-specific or suffer from a patient-induced bias, and the sampling time also correlates to different stages in the disease’s evolution due to the treatment’s effects (Figs G and H in S1 Appendix). Thus, it complexifies the deconvolution and removal of the batch effects while preserving the biological signal. We preprocessed the data with a log-normalization step using a total count of 10,000 as suggested by the authors.

Refer to S1 Appendix for details on the datasets.

## Results

### Clustering

**Fig 3:**
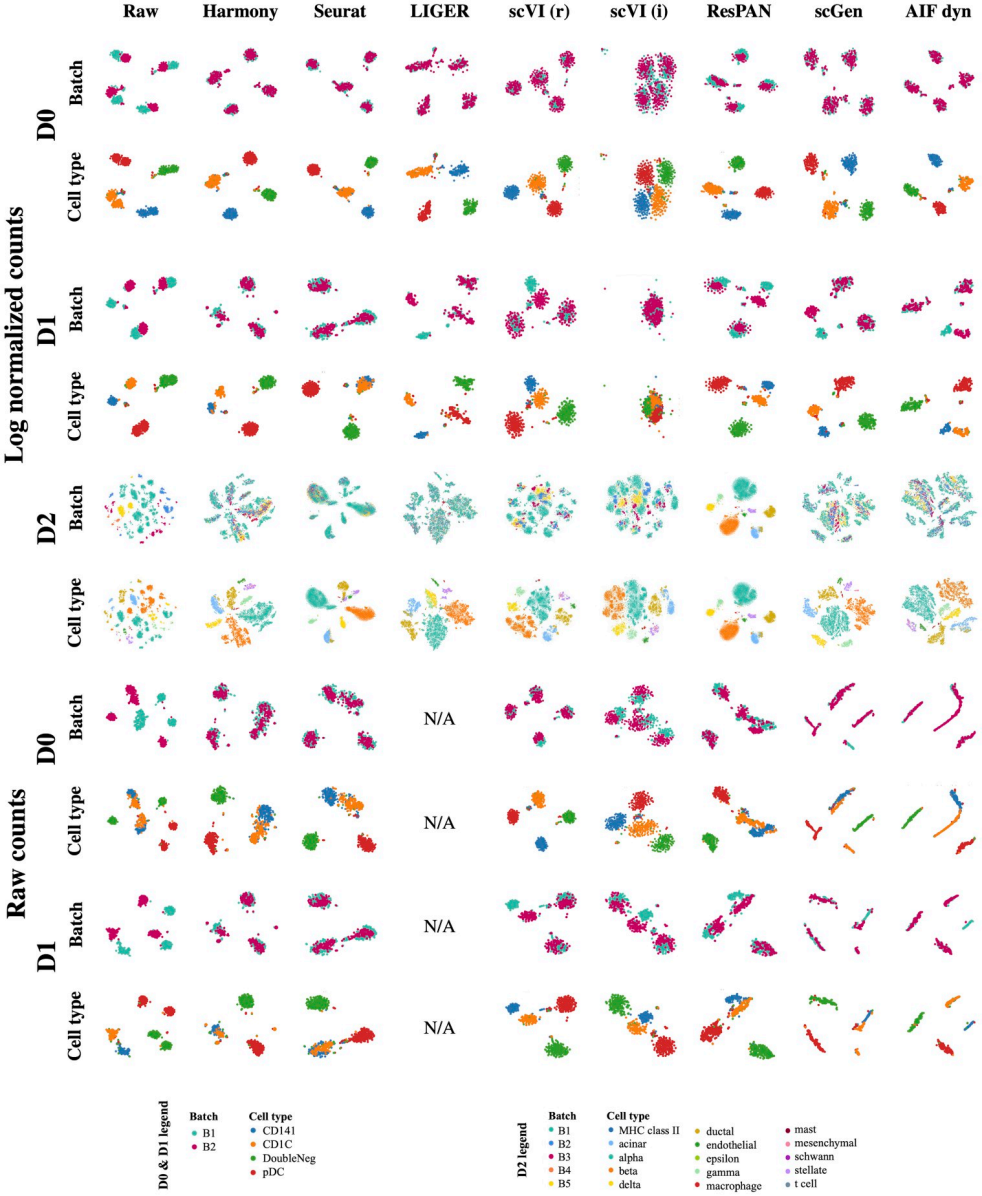
t-SNE visualizations of the original and batch-effect corrected data. The t-SNE is computed for the original data (1st column) and the methods’ corrected data (columns 2 to 9) on the three datasets’ log-normalized (rows 1 to 6) and raw counts (rows 7 to 10). The cells are colored with respect to their batch labels (odd lines) and cell type labels (even lines).

We selected the AIF dyn models based on each dataset’s maximum F1 ARI, F1 ASW, and F1 LISI scores (Table A in S3 Appendix). For scGen’s, Harmony’s, and LIGER’s training, we borrowed the optimal hyperparameters’ values inferred in [33]. For scVI (i), scVI (r), and ResPAN, the hyperparameters recommended by the authors (Section 2 in S3 Appendix) were used. We excluded LIGER for the raw Datasets, as a normalization step is necessary to run this method. We represented the t-SNE visualization of the corrected data for each model and each dataset in Fig 3. The clustering metrics on the full dataset are reported in Table 1. The models’ metrics’ robustness is outlined in Fig A in S5 Appendix.

**Table 1:** Comparison of the methods based on the clustering metrics computed on the full datasets.

	**Dataset 0**									**Dataset 1**								
	ARI			ASW			LISI			ARI			ASW			LISI		
	CT	1-B	F1	CT	1-B	F1	CT	B	F1	CT	1-B	F1	CT	1-B	F1	CT	B	F1
Harmony	0.64	1.00	0.78	0.45	1.00	0.62	1.02	1.81	0.80	0.70	1.00	0.82	0.44	0.99	0.61	1.01	1.33	0.47
Seurat	0.70	1.00	0.83	0.52	1.00	0.69	1.01	1.88	**0.84**	0.70	1.00	0.83	0.43	1.00	0.60	1.01	1.23	0.36
scVI (r)	0.79	1.00	0.88	0.65	1.00	**0.79**	1.00	1.51	0.63	0.87	1.00	**0.93**	0.61	0.95	**0.75**	1.00	1.10	0.18
scVI (i)	0.81	1.00	**0.89**	0.47	0.98	0.64	1.00	1.35	0.48	0.80	1.00	0.89	0.51	0.96	0.66	1.00	1.14	0.24
ResPAN	0.64	1.00	0.78	0.45	0.99	0.62	1.01	1.54	0.65	0.76	1.00	0.87	0.52	0.97	0.68	1.00	1.05	0.10
scGen	0.63	1.00	0.77	0.44	0.98	0.61	1.06	1.74	0.76	0.76	1.00	0.86	0.49	0.98	0.65	1.02	1.26	0.40
AIF dyn	0.81	1.00	**0.89**	0.56	0.99	0.72	1.02	1.88	**0.84**	0.83	1.00	0.91	0.62	0.95	**0.75**	1.01	1.48	**0.60**

	**Dataset 0 norm log**									**Dataset 1 norm log**								
	ARI			ASW			LISI			ARI			ASW			LISI		
	CT	1-B	F1	CT	1-B	F1	CT	B	F1	CT	1-B	F1	CT	1-B	F1	CT	B	F1
Harmony	0.78	1.00	0.87	0.64	1.00	0.78	1.00	1.93	0.86	0.79	1.00	0.88	0.58	0.96	0.72	1.00	1.60	0.69
LIGER	0.75	1.00	0.86	0.62	1.00	0.77	1.00	1.97	**0.88**	0.80	1.00	0.89	0.63	0.87	0.73	1.00	1.33	0.47
Seurat	0.79	1.00	0.88	0.66	1.00	0.79	1.00	1.85	0.82	0.70	1.00	0.83	0.47	1.00	0.64	1.00	1.23	0.56
scVI (r)	0.80	1.00	0.89	0.60	1.00	0.75	1.00	1.80	0.80	0.79	1.00	0.88	0.54	0.97	0.69	1.00	1.30	0.44
scVI (i)	0.79	1.00	0.88	0.45	0.98	0.62	1.00	1.75	0.77	0.51	1.00	0.68	0.25	0.98	0.40	1.02	1.71	**0.74**
ResPAN	0.80	1.00	0.89	0.69	1.00	**0.82**	1.00	1.60	0.68	0.66	1.00	0.79	0.34	0.97	0.50	1.02	1.36	0.50
scGen	0.77	1.00	0.87	0.61	1.00	0.76	1.00	1.94	0.86	0.82	1.00	**0.90**	0.64	0.95	**0.76**	1.00	1.49	0.61
AIF dyn	0.82	1.00	**0.90**	0.66	1.00	0.80	1.00	1.95	0.87	0.81	1.00	0.89	0.57	0.98	0.72	1.00	1.66	0.73

	**Dataset 2 norm log**									**Average F1 score**				
	ARI			ASW			LISI			D0		D1		D2
	CT	1-B	F1	CT	1-B	F1	CT	B	F1	raw	norm	raw	norm	norm
Harmony	0.95	0.99	0.97	0.45	1.04	0.63	1.00	1.50	0.22	0.63	0.72	0.26	0.88	0.54
LIGER	0.86	1.00	0.92	0.45	1.06	0.63	1.01	2.36	**0.50**	—	0.58	—	0.65	0.45
Seurat	0.94	0.99	0.96	0.54	1.03	0.71	1.00	1.61	0.26	0.35	0.72	0.15	0.57	0.71
scVI (r)	0.95	0.95	0.95	0.51	1.07	0.69	1.00	1.08	0.04	0.78	0.69	0.66	0.57	0.44
scVI (i)	0.95	0.94	0.94	0.39	1.14	0.58	1.00	1.04	0.02	0.38	0.37	0.40	0.33	0.12
ResPAN	0.93	0.98	0.95	0.52	1.06	0.70	1.00	1.59	0.26	0.20	0.60	0.30	0.33	0.63
scGen	0.96	0.99	**0.98**	0.55	1.06	**0.72**	1.00	1.49	0.22	0.26	0.72	0.41	0.86	**0.80**
AIF dyn	0.95	0.99	0.97	0.46	1.06	0.65	1.00	1.68	0.29	**0.87**	**0.95**	**0.92**	**0.94**	0.62

The metrics are evaluated on the corrected data’s t-SNE embeddings for the small datasets’ (Datasets 0 and 1) raw and log-normalized counts and UMAP embeddings for Dataset 2. Each metric is computed for the cell type purity (CT), the batch mixing (B), and combining both criteria (F1). The average F1 score is the average of the min-max standardized F1 ARI, ASW, and LISI scores. Top **1** performing methods are highlighted in **bold**.

The AIF dyn model outperforms state-of-the-art methods on the raw counts, significantly improving the F1 ARI, ASW and LISI metrics (first or second for both datasets), corresponding to the highest average F1 scores. Although scVI models obtain a similar cell type purity performance, they provide inferior batch mixing abilities. It proves our method’s efficiency in removing batch effects while preserving the biological signal, even in the most complex use cases where the batch effects are higher than the biological difference between cell types (Dataset 0) or with very similar batch-specific cell types under considerable batch effects (Dataset 1). Moreover, on the pre-processed version of Dataset 0, the AIF dyn model surpasses all methods’ F1 ARI while having the second-best F1 ASW and F1 LISI metrics, yielding the best average F1 score. On the Dataset 1 norm log, AIF dyn ranks second in F1 ARI and F1 LISI and third in F1 ASW, corresponding to the best average F1 score, due to its superior batch mixing ability while preserving a cell type purity similar to the other methods.

For the human pancreas cells datasets (Dataset 2 norm log), the AIF dyn model ranks second in F1 ARI and F1 LISI respectively, and fourth overall considering the average F1 score, closely behind ResPAN. scGen and Seurat’s higher cell type ASW comes from the supervised normalization (scGen) or the optimization on a clustering objective (anchors’ gene expression matching for Seurat), which forces the model to yield well-structured clusters (separated and compact) but erases some true biological variability coming from the cells’ heterogeneity within a cell type and even between cell types: some stellate cells are mixed with ductal cells in Seurat’s corrected data. We note the same phenomenon for LIGER, which merged epsilon and delta cells. For ResPAN, the higher cell type ASW is due to the already dimensionally reduced space (top 2,000 HVGs) and a Deep Learning gene expression matching strategy learned by the auto-encoder, thus yielding tighter clusters. Moreover, Seurat’s high clustering results are not representative of its overall performance, mainly resulting from a good separation of the highly-represented clusters (e.g., alpha, beta, delta, ductal, and epsilon cells) but not reflecting its poor performance on the small clusters (macrophage, stellate, endothelial, and ductal cells are overlapping), since the clusters’ size is not accounted for in the metrics. Although scGen’s supervision enables the preservation of small clusters (e.g., mesenchymal, macrophage, mast, etc.), it comes at the cost of a prior annotation of the cell types, which is often unavailable and highly time-consuming.

Regarding the robust models’ clustering evaluation pipeline (Fig A in S5 Appendix), we observe the same ranking as previously, with low standard deviations in the distributions for all use cases, except for some models’ ARI on Dataset 2 norm log. Indeed, the clustering algorithm sometimes fails to output relevant clusters due to the very imbalanced cell types and the sampling process not being designed accordingly. Thus, one should consider the full dataset’s metrics for this use case to alleviate the bias induced by the clustering algorithm’s failures.

Overall, AIF dyn provides the best results, using both the classic or the robust clustering evaluation pipeline, in 4 out of 5 cases. Besides, it ranks fourth on the last dataset, closely behind ResPAN, with similar cell type purity and better batch mixing. It proves AIF dyn’s great ability to preserve the biological signal while aligning the batches in all scenarios.

### Differential expression

**Fig 4:**
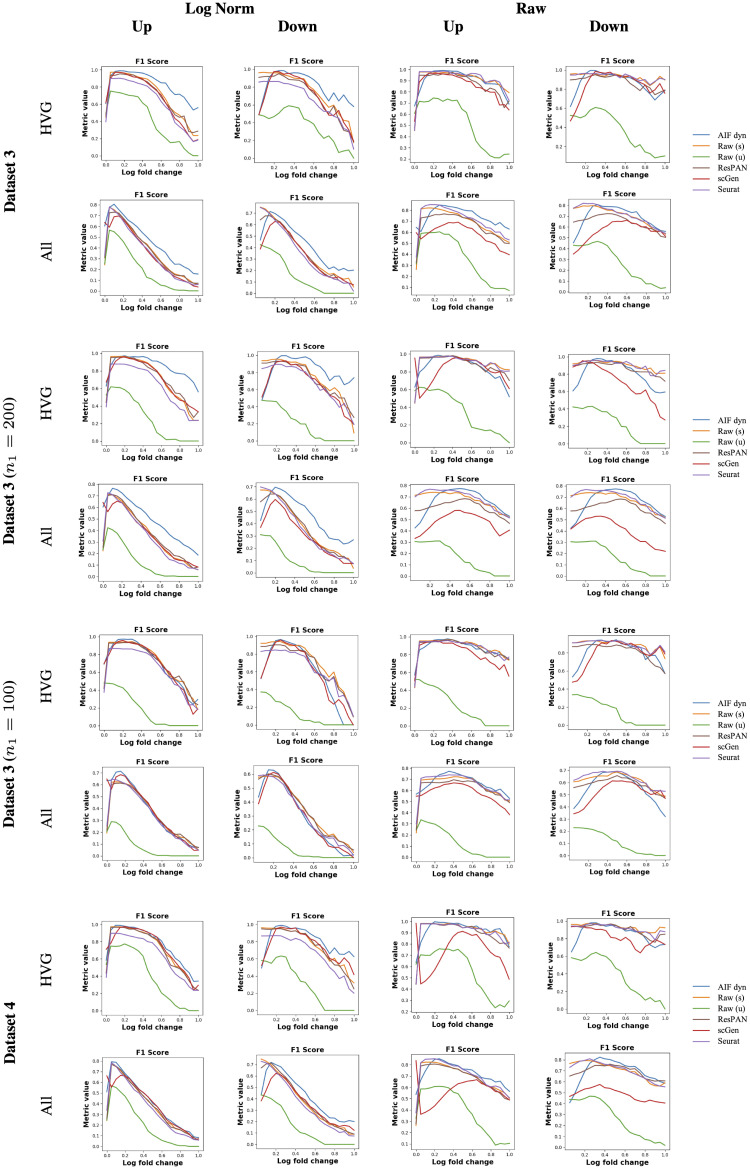
Evolution of the up and down-regulated DEGs’ F1 score with the log-fold-change threshold. The results are shown for the highly variable genes (’HVG’) and all genes (’All’). The reported log-fold-change values are in base 2.

Only Seurat, ResPAN, scGen, and AIF dyn were considered for this section. Harmony’s, LIGER’s, and scVI’s batch-effect corrections were performed in an embedded space, limiting their use to clustering tasks solely. ResPAN also reduces the feature space’s dimension by extracting the top 2,000 HVGs in either of the two batches, impeding the DE analysis. To enable the inclusion of ResPAN in the comparison, we completed the gene expression with the raw data for the 3,000 missing genes, considering that the batch effects mainly resided in the genes extracted by ResPAN. We compared their differential expression results to the uncorrected data using unsupervised labels (Raw (u)) produced by K-Means clustering or the supervised labels (Raw (s)). All models’ corrected data resulted in an almost perfect clustering (Table B in S6 Appendix). Hence, the differences in performance are inherent to the models’ ability to preserve cell types’ gene expression. To investigate the models’ ability to conserve the DEGs for all levels of expression ratios, we computed the F1 score for the up and down-regulated genes in cell type 1, using different log-fold-change thresholds for genes’ filtering. The F1 score’s evolution is depicted in Fig 4. We reported the corresponding Area Under the Curve (AUC) in Table 2 to provide an overall score accounting for all thresholds and facilitate the models’ comparison.

**Table 2:** Comparison of the methods’ differentially expressed genes’ AUC score for the simulated datasets (all versions of Dataset 3 and Dataset 4).

	**Dataset 3**								**Dataset 3** (*n*_1_ = 200)							
	**Log Norm**				**Raw**				**Log Norm**				**Raw**			
	HVG		All		HVG		All		HVG		All		HVG		All	
	↑	↓	↑	↓	↑	↓	↑	↓	↑	↓	↑	↓	↑	↓	↑	↓
**Raw (s)**	0.72	0.75	0.38	0.38	0.92	**0.92**	0.70	0.70	0.72	0.75	0.37	0.37	**0.92**	**0.89**	0.68	0.67
**Raw (u)**	0.41	0.37	0.19	0.15	0.53	0.38	0.37	0.27	0.26	0.19	0.12	0.10	0.36	0.23	0.23	0.16
**ResPAN**	0.70	0.71	0.38	0.37	0.88	0.87	0.67	0.64	0.73	0.73	0.38	0.37	0.89	0.86	0.66	0.60
**scGen**	0.68	0.70	0.36	0.35	0.86	0.84	0.59	0.56	0.72	0.67	0.36	0.32	0.81	0.72	0.56	0.48
**Seurat**	0.64	0.66	0.36	0.35	**0.93**	**0.92**	0.73	**0.71**	0.63	0.68	0.35	0.36	0.91	**0.89**	0.70	**0.68**
**AIF dyn**	**0.84**	**0.81**	**0.47**	**0.44**	0.91	0.86	**0.75**	0.69	**0.86**	**0.83**	**0.50**	**0.47**	0.86	0.81	**0.73**	0.66
	**Dataset 3** (*n*_1_ = 100)								**Dataset 4**							
	**Log Norm**				**Raw**				**Log Norm**				**Raw**			
	HVG		All		HVG		All		HVG		All		HVG		All	
	↑	↓	↑	↓	↑	↓	↑	↓	↑	↓	↑	↓	↑	↓	↑	↓
**Raw (s)**	**0.72**	**0.72**	0.37	**0.35**	**0.90**	**0.88**	0.65	0.61	0.73	0.76	0.38	0.38	**0.93**	**0.92**	0.70	**0.70**
**Raw (u)**	0.17	0.13	0.08	0.06	0.22	0.16	0.13	0.11	0.39	0.30	0.20	0.15	0.56	0.39	0.40	0.27
**ResPAN**	**0.72**	0.68	0.37	0.34	0.88	0.82	0.63	0.58	0.72	0.76	0.39	0.39	0.91	0.89	0.69	0.68
**scGen**	0.69	0.61	**0.38**	0.31	0.83	0.79	0.58	0.52	0.74	0.76	0.39	0.37	0.73	0.83	0.56	0.50
**Seurat**	0.65	0.64	0.35	0.32	0.89	**0.88**	0.66	**0.63**	0.65	0.67	0.36	0.36	0.92	**0.90**	0.72	**0.70**
**AIF dyn**	0.71	0.60	**0.38**	0.32	0.88	0.81	**0.67**	0.56	**0.77**	**0.81**	**0.42**	**0.43**	**0.93**	0.86	**0.73**	0.69

The statistical test is performed using raw or log-normalized counts, on the highly variable genes (HVG) or all genes (All), for the up (↑) and down-regulated (↓) DEGs. The F1 score is computed at different log-fold-change thresholds, and the corresponding Area Under the Curve (AUC) is reported in this table. Top **1** performing methods are highlighted in **bold**.

Contrary to what is stated in [33], the batch effects in the simulated data are not high and complex enough to confound the supervised DEG detection, leading to high F1 scores, especially for the raw counts. Thus, the supervised uncorrected DEGs’ results would constitute a good sanity check for the batch-effects correction methods’ preservation of the biological signal. Running the DEG detection pipeline in an unsupervised fashion, which is usually done in practice, resulted in a considerable drop in performance due to one cluster mixing two cell types (Fig A in S6 Appendix). AIF dyn outperforms the other models on the log-normalized counts, most commonly used for DE analysis,for all use cases, except the extremely imbalanced cell types (Dataset 3 (*n*_1_ = 100)) where it still yields top-ranked results. From log_2_*FC* = 0.2, it surpasses all methods, even the supervised uncorrected DEG detection, on the log-normalized counts considering all genes or only HVGs. This proves that AIF dyn successfully refined the cell types’ expression profiles by removing noisy batch effects, mainly on the non-HVGs, which are the trickiest. Although Seurat yields slightly greater results on the raw counts in Table 2, reaching the performance of the supervised uncorrected DEGs detection (Raw (s)), AIF dyn is a close second. Besides, it surpasses both for *log*_2_*FC* ≥ 0.25, particularly on non-HVGs with a high fold-change. Overall, AIF dyn best preserves the biological difference between cell types in the relative gene expression (i.e., log-normalized counts), which is the common practice, while providing great results on the intrinsic gene expression (i.e., raw counts), even when dealing with very imbalanced cell types and high dropout factors. Note that ResPAN’s results are similar to the supervised raw data since more than half of the genes were left uncorrected and correspond to the Raw (s) model. The slight deterioration observed comes from the batch-effect corrected genes but is diluted by the other genes. Thus, we performed the same analysis on ResPAN’s genes subset only (Fig D in S6 Appendix) to evaluate the quality of ResPAN’s batch-effect correction solely.

### Application to Acute Myeloid Leukemia (AML)

**Fig 5:**
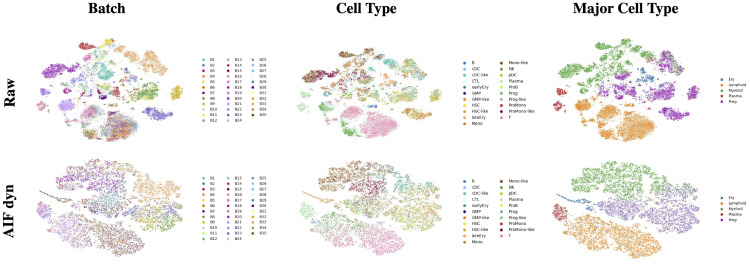
t-SNE visualizations of the original and batch-effect corrected data for the AML dataset. The t-SNE is computed for the original data (top row) or AIF’s batch-effect-corrected data (bottom row) using a prior PCA. The cells are colored by batch label (left), cell type label (middle), or major cell type label (right).

To demonstrate AIF dyn’s applicability to more complex real-world clinical applications, we investigated a scRNA-seq dataset proposed by [40], which is a cohort of 16 AML-diagnosed patients with multiple time points (at diagnosis and after treatment). It consists of 30,334 cells from bone marrow aspirates, comprising 35 batches defined as patient × time point, and 21 cell types spanning the hematopoiesis’s differentiation tree. This dataset contains both healthy and malignant cells, the latter being denoted with a “-like” suffix. First, we assessed the quality of AIF dyn’s batch-effect correction with the clustering metrics presented in the benchmark section. Those metrics are computed at different resolutions: using the authors’ cell types annotations or the major cell types, i.e., the differentiation branch they belong to. The t-SNE visualizations of the original and corrected data at the different resolutions are represented in Fig 5. The corresponding clustering metrics are reported in Table 3. AIF dyn’s batch-effect correction improves all clustering metrics except the cell type LISI, which has deteriorated due to the noisy immature cells. The batch-mixing-related metrics are significantly boosted while corresponding to enhanced cell-type-purity metrics, especially for the major cell types. This proves the benefits of batch-effect correction and highlights our model’s great abilities even when dealing with a complex setting. The lower results on the fine-grained resolution are caused by the immature cells’ cluster and the continuous distribution, which motivated the model’s evaluation on the more coarsened resolution. Indeed, the immature cells are indistinguishable for most patients, yielding only one cluster for all of them, thus hampering the clustering results. Besides, hematopoiesis has recently been viewed as a continuous process rather than a discrete one [46, 47], which hinders the clustering task since the partitioning of a continuum is arbitrary.

**Table 3:** Comparison of the clustering results on the AML dataset.

	Cell type									Major cell type								
	ARI			ASW			LISI			ARI			ASW			LISI		
	CT	1-B	F1	CT	1-B	F1	CT	B	F1	CT	1-B	F1	CT	1-B	F1	CT	B	F1
**Raw**	0.40	0.59	0.48	-0.04	1.01	-0.08	1.46	1.23	0.01	0.33	0.59	0.41	0.09	1.01	0.17	1.00	1.23	0.01
**AIF dyn**	**0.42**	**0.81**	**0.55**	**-0.03**	**1.21**	**-0.06**	2.27	**3.32**	**0.13**	**0.68**	**0.82**	**0.74**	**0.22**	**1.21**	**0.37**	1.01	**3.38**	**0.13**

The metrics are based on Louvain clustering on the t-SNE embeddings with a prior PCA, using either the cell type (left) or the major cell type (right) labels. Each metric is computed for the cell type purity (CT), the batch mixing (B), and combining both criteria (F1). The best results are highlighted in bold.

**Fig 6:**
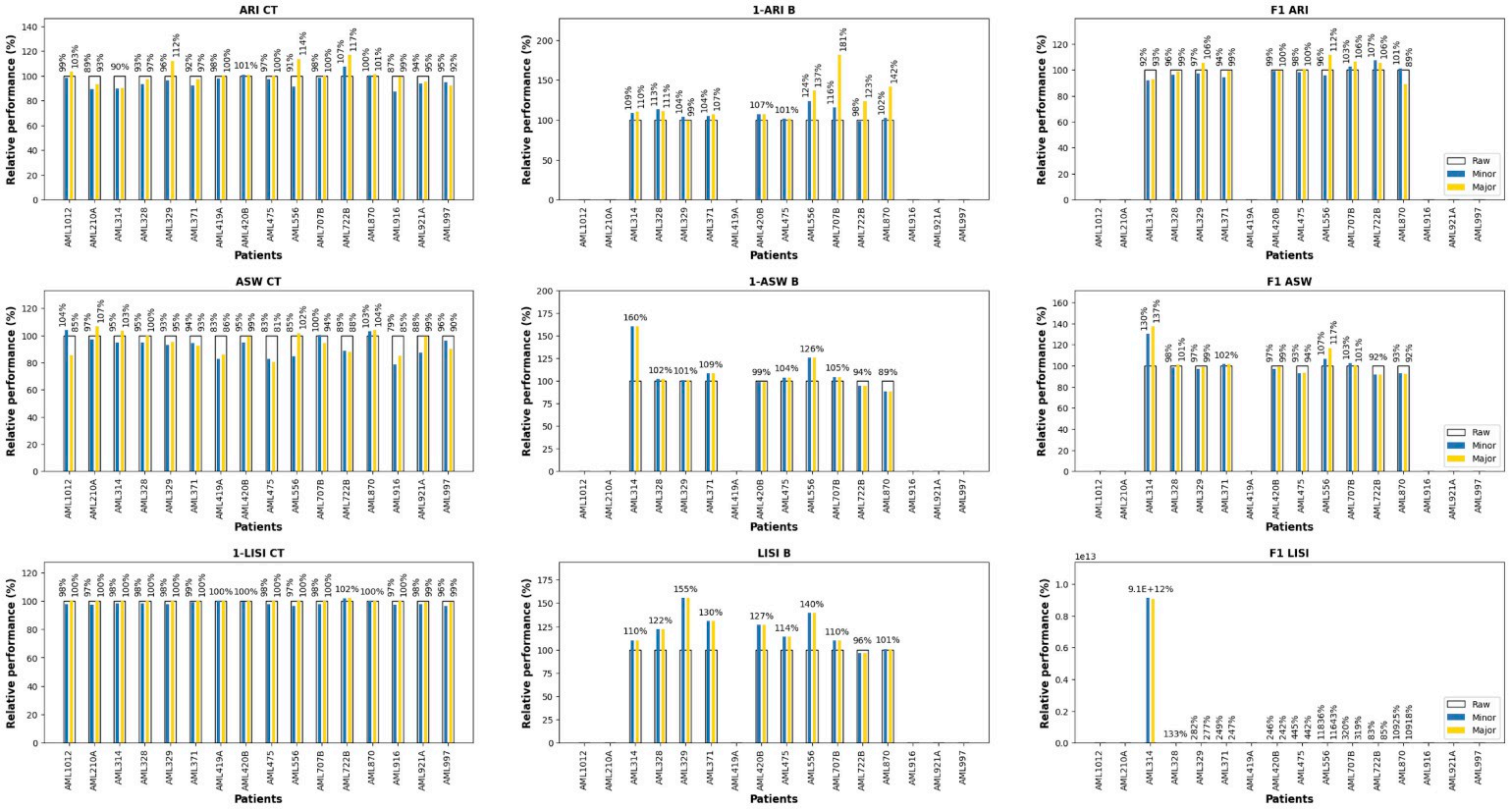
Clustering relative performance per patient. The clustering relative performance corresponds to the ratio between the corrected and original data’s performance for each clustering metric priorly scaled. Those metrics are based on Louvain clustering on each patient’s t-SNE embeddings with a prior PCA, using either the cell types (blue) or the major cell types (yellow). Each metric is computed for the cell type purity (CT), the batch mixing (B), and combining both (F1).

To further assess the batch-effect correction’s quality, we performed the clustering evaluation pipeline on each patient individually. Indeed, the batch effects induced by the time points are insignificant for most patients, except for patient AML707B. Thus, the clustering on the patient’s original data should capture all the biological information possibly retrievable to discriminate between the cell types, constituting reasonable upper bounds of the cell type purity metrics. We computed the corrected data’s clustering relative performance compared to the original data, i.e., the ratio between the corrected and original data’s metrics, for each patient and at both resolutions (fine-grained and coarsened). Note that the batch and F1 metrics are only computed for multiple-time-points-patients. All metrics are priorly scaled so that they range between 0 and 1, except the F1 LISI for which the batch and cell type metrics were already scaled. We reported the average relative clustering performance across patients in Table 4, and detailed the per-patient results in Fig 6. Note that the large average F1 LISI ratios are due to AML314’s batch LISI being close to 1 in the original data. Overall, AIF dyn’s batch-effect correction significantly improves all batch-mixing-related metrics by at least 8% on average, while preserving most of the cell type purity (≥92% on average), resulting in higher F1 metrics, except the F1 ARI based on the fine-grained resolution. These trends are even accentuated for the major cell types where all the metrics are improved compared to the original data, except the cell type ASW due to the data’s continuous distribution and the immature cells’ noisiness. This implies that the batch-effect correction even refined some patients’ cell distribution (patients AML1012, AML329, AML420B, AML556, AML722B, and AML870) by learning from the other patients’ cell distribution. Altogether, those results confirm AIF dyn’s ability to efficiently correct batch effects while preserving most of the biological signal.

**Table 4:** Average per-patient clustering relative performance.

	ARI			ASW			LISI		
	CT	1-B	F1	CT	1-B	F1	1-CT	B	F1
**Cell Type (%)**	95.5	107.9	98.4	92.3	108.9	101.1	98.3	120.4	9.1*e*11*
**Major Cell Type (%)**	100.8	121.9	100.9	94.5	108.9	103.3	100.0	120.4	9.1*e*11*

The clustering relative performance corresponds to the ratio between the corrected and original data’s performance for each clustering metric priorly scaled. Those metrics are based on Louvain clustering on each patient’s t-SNE embeddings with a prior PCA, using either the cell types (top) or the major cell types (bottom), and are then averaged across all patients. Each metric is computed for the cell type purity (CT), the batch mixing (B), and combining both (F1). Note that the large F1 LISI ratios (*) are due to AML314’s LISI batch close to 1 in the original data.

## Discussion

In the clustering study, we focused our analysis on three datasets that covered a variety of complex real-world use cases: low signal-to-noise ratio, imbalanced and batch-specific cell types combined or not with imbalanced multi-batches. By investigating the models’ performance on both the raw and log-normalized counts, we highlighted that the normalization step helps improve the resulting batch-effect correction for all methods from the benchmark, as it reduces the variability between cells but also leads to an alteration of the biological signal, which hampered the convergence of the AIF dyn models and the clustering metrics. However, the AIF dyn models were the most consistent across the datasets’ versions.

We benchmarked our method on a clustering task against state-of-the-art algorithms, some of which were explicitly designed for clustering. Indeed, four methods rely on a clusters’ matching strategy: a supervised one for scGen and unsupervised ones for Harmony, LIGER, and Seurat, resulting in tight cell distributions, which could be unrealistic and lead to the loss of the cells’ biological heterogeneity. Even though our method is task-agnostic, we successfully outperformed these methods in four out of five cases, especially without prior normalization. It raises excellent hopes as the AIF dyn was not optimized toward a clustering task. A parallel to the other methods’ bias towards clustering would be adding a supervised or unsupervised clustering loss to the overall encoder’s objective, as performed in [48], improving the previous clustering metrics. Regarding the other deep learning methods (scVI and ResPAN), AIF dyn provides superior batch mixing and biological preservation on the human blood datasets and compares to ResPAN on the human pancreas dataset, where scVI fails to mix the batches with a LISI batch close to 1. Although we selected a better-suited embedding algorithm (t-SNE or UMAP), it still induced a bias in the metrics, as they are computed in an embedded space. Moreover, K-Means’s performance wrongly penalized some models, as it is unsuited for all data distributions, and Louvain struggled with small clusters. Other constructions of the initial graph and better tuning of its hyperparameters could provide a fairer comparison. A more detailed discussion on the metrics’ biases can be found in S5 Appendix.

To address the clustering task’s bias, we benchmarked the models on differential expression analysis using simulated datasets. The AIF dyn model best preserved the relative gene expression within cells and between cell types, as it outmatched all models on the log-normalized counts, which is the most commonly used in DEG detection, on three out of four datasets. It also improved the cell types’ intrinsic signal (raw counts) for highly differentiated genes, ranking first for log_2_*FC* ≥ 0.25. Note that a double-dipping issue arises from using the same genes for clustering and DE analysis, which could be addressed by performing DEG detection and clustering simultaneously with an NN model or on different modalities. The final objective after batch-effect correction can be diverse, such as survival analysis, treatment effect prediction, distribution of housekeeping genes, etc. Other performance metrics derived from those tasks should be used to fairly evaluate the model’s ability to retain all relevant information for any downstream analysis. Another possibility would be to assess the method performance by aligning scATAC-seq and scRNA-seq data in the spirit of [49]. Still, it is unclear how the methods would handle such different sources.

Regarding scalability, AIF dyn ranks among the best models in memory usage, thanks to its stochastic optimization (Fig A in S3 Appendix). AIF dyn requires longer training than the other deep learning methods (scGen, scVI, and ResPAN) since it does not answer the same problem. scGen and scVI learn the cell distributions tainted by the batch effects, resulting in a poorer batch mixing ability for scVI and requiring a supervised normalization post-training for scGen, often impossible due to the absence of annotations. ResPAN only works on a gene subset (2,000 HVGs in either batch) and only learns how to project the cells onto one reference batch. Instead, AIF dyn learns all batches’ distributions simultaneously, thus enabling the projections onto all batches without re-training the model contrary to ResPAN. Besides, its more complex formulation allows a better preservation of the biological signal in the DEG analysis. The increased complexity induced by the two additional networks in AIF dyn leads to higher computational time than a CVAE but only affects the training step and improves the model’s convergence: the MSE was lower after 1000 epochs than for the corresponding CVAE resulting in higher metrics (Figs A and B in S4 Appendix). We believe the training time could be significantly decreased by better-suited losses’ normalization and weighting strategies since some losses (MSE, classification, and projection constraint) are quickly stagnating (Fig B in S3 Appendix).

## Conclusion

We proposed a novel method based on deep learning to correct batch effects in scRNA-seq datasets. Although our AIF model is task-agnostic, it outperforms or compares to state-of-the-art clustering-biased algorithms and deep-learning models on complex use cases. Besides, it is the least sensitive to normalization, as it equivalently works with raw and normalized counts. In addition, our approach does not rely on prior knowledge of the cell types, contrary to scGen. It is adapted for any downstream analysis since it corrects scRNAseq data in the original space, contrary to Harmony, LIGER, and scVI. It also enables the immediate integration of new samples with a consistent performance (Table E in S4 Appendix), while Harmony, LIGER, and Seurat cannot, conferring a great advantage to our method as it suits more diverse use cases. Finally, our architecture enables a flexible batch-effect correction that is not the same for each gene but is adapted to each gene set embedded in the latent features, translating into great differential expression results. Indeed, AIF dyn surpasses the other methods on the log-normalized counts, indicating better preservation of the relative gene expression within cells, and yields top-ranked results on the raw counts with higher preservation of the highly differentially expressed genes (i.e., with a high fold-change), even if not highly variable. Finally, we illustrated AIF dyn’s batch-effect correction’s quality on a more complex setting crystallized by the AML dataset, where most of the underlying biological information was preserved for each patient while aligning the batches, resulting in higher clustering metrics in the pooled dataset.

## Supporting information

S1 Appendix Datasets’ details. This appendix details the datasets’ characteristics and cell distribution.
(PDF)

S2 Appendix Evaluation procedure. This appendix delves into the different steps of the evaluation pipeline employed in this study to benchmark the models on the clustering and the differential expression analysis tasks.
(PDF)

S3 Appendix Training details. This appendix presents the AIF dyn architecture’s and training’s details. It also describes the training hyperparameters used for each model in the benchmark and their training time and memory usage.
(PDF)

S4 Appendix AIF dyn’s results. This appendix summarizes the AIF dyn’s main results regarding the robustness of the multi-sample batch-effect correction method, the influence of the losses’ weights, the benefits of the dynamic ratio, the projection constraint, and delaying the auxiliary and GAN networks. We also investigated the model’s ability to generalize to unseen samples compared to scGen.
(PDF)

S5 Appendix Models’ clustering robustness and metrics’ biases. This appendix investigates the models’ clustering robustness and the biases induced in the metrics by the embedding and clustering algorithms’ performance.
(PDF)

S6 Appendix DE analysis results. This appendix presents the clustering results before the DE analysis and delves into the DE results.
(PDF)
