# Supplementary material for: A novel batch-effect correction method for scRNA-seq data based on Adversarial Information Factorization
Source: PLoS Comput Biol. 2024 Feb 22;20(2):e1011880. doi: 10.1371/journal.pcbi.1011880 (PMC10914288; doi:10.1371/journal.pcbi.1011880)
Supplement: S1 Appendix — This appendix details the datasets’ characteristics and cell distribution. (PDF) [file pcbi.1011880.s001.pdf]

S1 Appendix: Datasets' details

Lily Monnier[1], Paul-Henry Cournède[1,*]
1. MICS Laboratory, CentraleSupélec, Paris-Saclay University, Gif-sur-Yvette, France.
* corresponding author: paul-henry.cournede@centralesupelec.fr

1 Benchmark datasets

The data used to evaluate the models on the clustering task are available under accession numbers GSE80171 [1], GSE85241 [2], E-MTAB-5061 [3], GSE84133 [4] GSE83139 [5], and GSE81608 [6]. The simulation data for DE analysis are generated using Splatter [7] with the parameters listed in Table E. The data are available at:
https://github.com/JinmiaoChenLab/Batch-effect-removal-benchmarking [8].

Table A is an overview of the datasets' characteristics. Tables B–D outline the cell types' distribution across batches for Datasets 0 and 1, Dataset 2 and the simulated datasets, respectively. For Dataset 2, the train-test split distribution is also represented.

We represented the t-SNE visualization of each version of the datasets' counts in Figs A–C for Dataset 0, Dataset 1 and Dataset 2 respectively. We can observe the impact of the log-normalization: attenuation of the batch effects but deterioration of the biological signal with the creation of artifact clusters for the human blood datasets or the subdivision of the cell types even within the same batch for the human pancreas dataset.

Table A. Datasets' characteristics.

	**Datasets**						
	D0	D1	D2	D3	D3 ($n_1 = 200$)	D3 ($n_1 = 100$)	D4
number of cells	768	576	14,767	1,400	1,189	1,090	1,400
number of genes	26,593	26,593	15,636	5,000	5,000	5,000	5,000
batch-specific cell types		✓	✓				
imbalanced cell types		✓	✓	✓	✓	✓	✓
imbalanced batches			✓	✓	✓	✓	✓
multi-batches			✓				
simulation				✓	✓	✓	✓

Table B. Human blood dendritic cells datasets' cell types distribution [1].

	**Dataset 0**				**Dataset 1**			
	CD1c+	CD141+	pDC	DN	CD1c+	CD141+	pDC	DN
B1	96	96	96	96	0	96	96	96
B2	96	96	96	96	96	0	96	96

The dataset comprises four cell types across two batches generated by the same technology.

Table C. Human pancreas cells dataset's distribution.

	**Training set**					**Test set**				
	B1	B2	B3	B4	B5	B1	B2	B3	B4	B5
acinar	778	168	150	6	0	180	51	35	0	0
alpha	1842	655	699	145	698	484	157	187	45	188
beta	2039	356	208	95	376	486	92	62	16	96
delta	476	152	90	8	40	125	41	24	1	9
ductal	878	197	307	80	0	199	48	79	16	0
endothelial	218	18	14	0	0	34	3	2	0	0
epsilon	16	2	6	0	0	2	1	1	0	0
gamma	208	77	148	13	68	47	24	49	5	17
macrophage	47	0	0	0	0	8	0	0	0	0
mast	16	0	7	0	0	9	0	0	0	0
mesenchymal	0	67	0	25	0	0	13	0	2	0
MHC class II	0	0	4	0	0	0	0	1	0	0
Schwann	11	0	0	0	0	2	0	0	0	0
stellate	357	0	44	0	0	100	0	10	0	0
t cell	5	0	0	0	0	2	0	0	0	0

The dataset comprises 15 cell types across five batches generated by different technologies: inDrops for B1 [2], CelSeq2 for B2 [3], SMART-seq2 for B3 [4], and SMARTer for B4 [5] and B5 [6].

Table D. Simulated datasets' cell types distribution.

	**D3**		**D3** ($n_1 = 200$)		**D3** ($n_1 = 100$)		**D4**	
	CT 1	CT 2	CT 1	CT 2	CT 1	CT 2	CT 1	CT 2
B1	148	352	82	352	39	352	148	352
B2	263	637	118	637	62	637	263	637
Total	411	989	200	989	101	989	411	989

The dataset comprises two cell types (CT 1 and CT 2) across two batches (B1 and B2) with a small dropout factor (≈ 0.05) for D3 and a larger dropout factor for D4 (≈ 0.25).

Table E. Simulations' parameters.

	**D3**	**D4**
$P(CT_1)$	0.3	0.3
$P(CT_2)$	0.7	0.7
$P(DE_1)$	0.2	0.2
$P(DE_2)$	0.1	0.1
$P(down_1)$	0.3	0.3
$P(down_2)$	0.4	0.4
mean(DE)	0.5	0.5
std(DE)	0.2	0.2
dropout factor	0.05	0.25
dropout style	experiment	experiment

The simulated data are generated through Splatter using the above parameters. $P(CT_i)$, $P(DE_i)$ and $PE(down_i)$ are the probabilities of cell type, DEGs and down-regulated DEGs in cell type i, mean(DE), and std(DE) are the mean and standard deviation of the differential expression distributions.

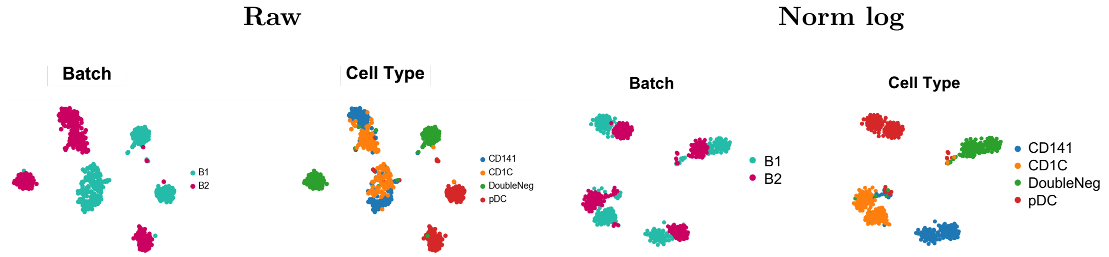

Figure A. t-SNE visualizations of the raw and pre-processed counts of Dataset 0. The cells are colored by either batch label (left) or cell type label (right). Note that for the log-normalized counts, a prior PCA is required.

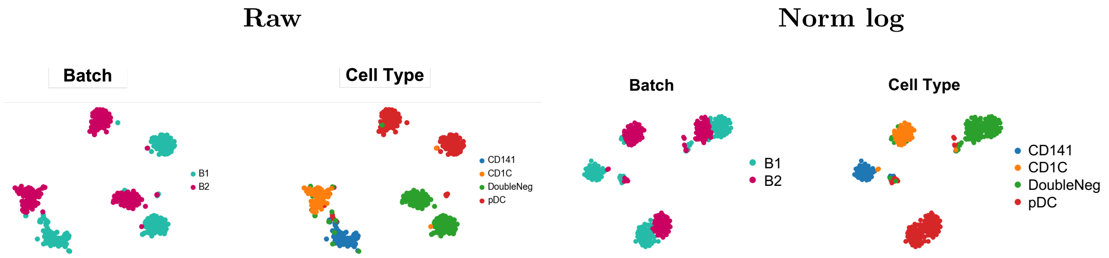

Figure B. t-SNE visualizations of the raw and pre-processed counts of Dataset 1. The cells are colored by either batch label (left) or cell type label (right). Note that for the log-normalized counts, a prior PCA is required.

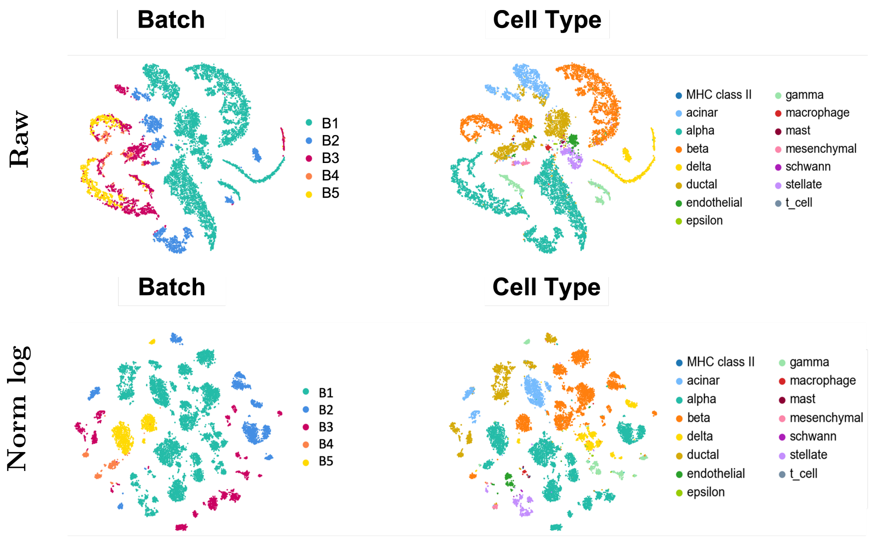

Figure C. t-SNE visualizations of the raw and pre-processed counts of Dataset 2. The cells are colored by either batch label (left) or cell type label (right). Note that for the log-normalized counts, a prior PCA is required.

2 Acute Myeloid Leukemia (AML) dataset

2.1 Presentation

This dataset constitutes a real-world clinical application on the effects of chemotherapy on a cohort of 16 AML-diagnosed patients [9]. The authors sequenced 30,334 cells from patients' biopsies (mixture of normal and tumorous cells) using scRNA-seq at different time points: at diagnosis (D0) and after undergoing chemotherapy ($Di, i > 0$) for most patients, spanning multiple time points potentially. Indeed, 11 of the 16 patients have longitudinal data, all receiving induction chemotherapy at D0, except AML328, whose treatment relies on Azacitidine and Venetoclax. The authors annotated 21 extremely unbalanced cell types, ranging from 104 for ProB cells to 6,381 for T cells. They named malignant cell types after the healthy ones they resemble, adding a *-like* suffix. The number of cells per patient or time point is also highly variable: from 104 cells for AML722B to 6,405 cells for AML328, with approximately 50% of the data corresponding to D0 and $< 1\%$ for some time points (e.g., D34, D37, D49, D97). In this scenario, both the patient and time point can be sources of batch effects. Indeed, the cells corresponding to the same time point and the same patient were sequenced together, so we defined the batches as patient $\times$ time for a total of 35 batches. The cells' repartitions across patients, cell types, and batches are depicted in Figs E and F and explicitly detailed in Table F. The dataset is represented with t-SNE visualizations in Fig D.

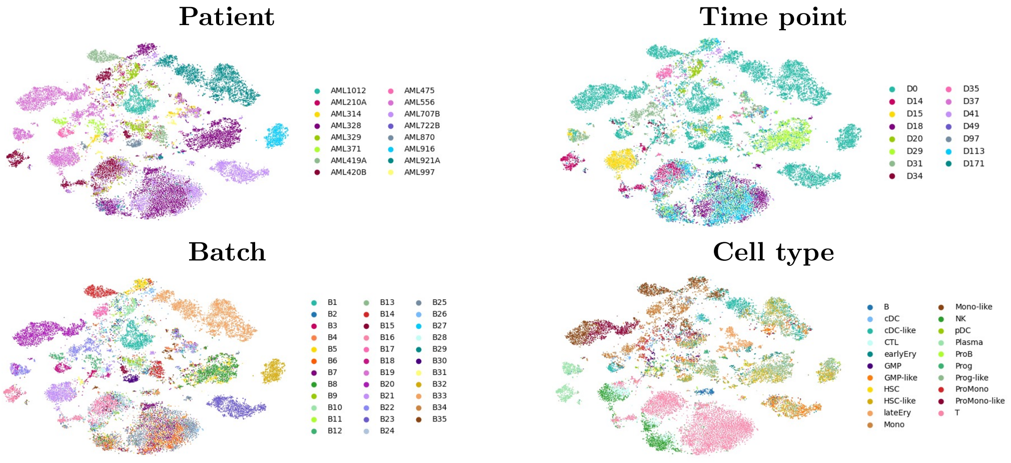

Figure D. t-SNE visualizations of the log-normalized counts of the AML dataset. The cells are colored by either patient (top-left), time point (top-right), batch label (bottom-left) or cell type label (bottom-right). Note a prior PCA is required for the t-SNE to work.

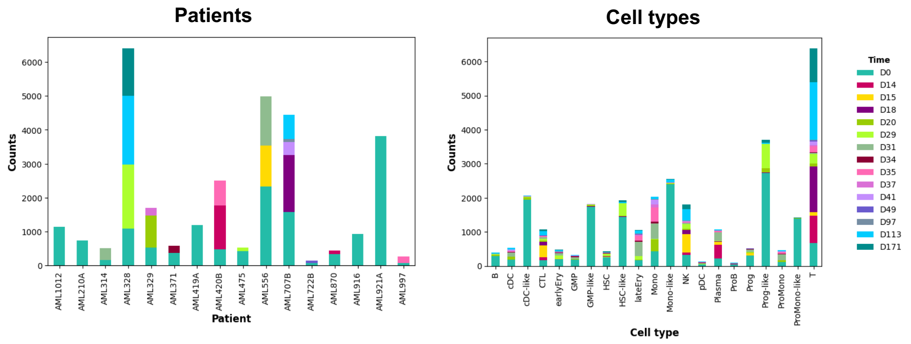

Figure E. Time points' repartition across AML dataset's cells. The histograms represent the time points' repartition in the total number of cells per patient (left) or cell type (right), the colors referring to the time points.

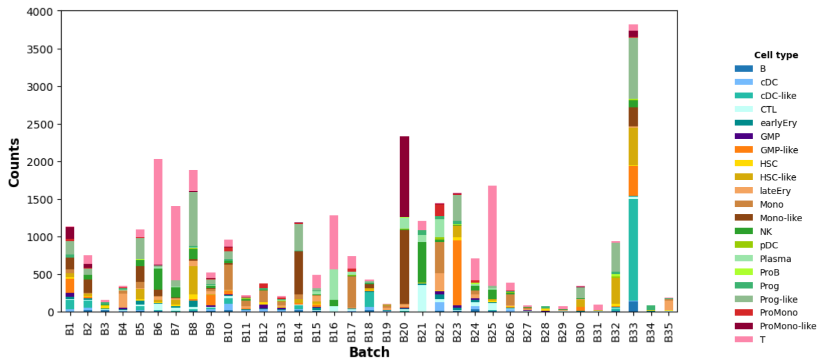

Figure F. Cell types' repartition across batches. The histogram represents the cell types' repartition in the total number of cells per batch, the colors referring to the cell types.

2.2 Batch effects' sources

To better grasp the sources of batch effects, first, we investigated the patient's bias in the cells' populations, leading to the subdivision of cell types due to patient-specific gene expression patterns. We illustrated this phenomenon in Fig G for both normal and malignant cell types. For healthy cells (top row), although the cell types should be similar across patients and yield a unique cluster, we observe multiple clusters corresponding to different patient labels, highlighting the patient's batch effects. Malignant cells can be patient-specific due to the mutations they acquire. Thus, some slight differences between patients may exist in these abnormal populations. However, the cells are very distant in the t-SNE visualization, which suggests that their biological differences are intertwined with batch effects.

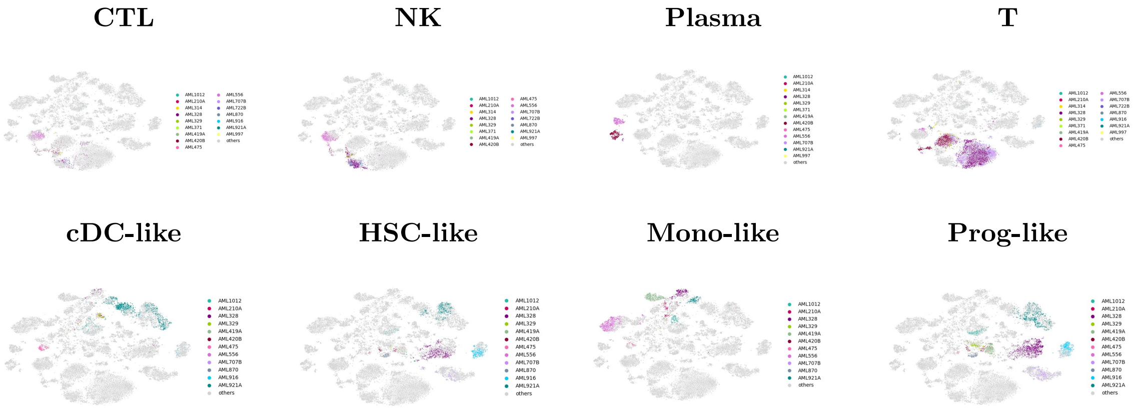

Figure G. Illustration of the patient's bias in healthy and malignant cells. The patient's bias is represented for different cell types corresponding to normal cells (top row) or malignant cells (bottom row). Each cell type's cell is colored by its patient label in the t-SNE visualization.

To expose the batch effects induced by the time points, we explored the cells' distribution of patient AML707B across time in Fig H. We observe a significant difference between D18's and D113's cell types, especially for T and Prog cells, which are split into two clusters each. Moreover, cells sampled at the diagnosis time cluster almost all together. Although they correspond to malignant cells for the majority, they should overlap with the other time points' tumorous cells since they all belong to the same individual. Nonetheless, the time points also embed some actual biological variation corresponding to the effects of chemotherapy (or treatment for AML328), which mainly affects the cells' mixture by suppressing malignant cells (cDC-like, GMP-like, HSC-like, Mono-like,

Table F. AML-diagnosed patients dataset's cells distribution.

Batches			Cell types																				
			B	cDC		CTL	earlyEry	GMP		HSC		lateEry	Mono		NK	pDC	Plasma	ProB	Prog		ProMono		T
Patients	Time	Batch		N	T			N	T	N	T		N	T					N	T	N	T	
AML1012	D0	B1	0	32	122	13	33	51	190	21	51	0	49	152	3	0	1	2	39	183	22	158	14
AML210A	D0	B2	25	24	94	16	14	6	23	3	35	0	8	182	56.0	0	3	1	5	79	12	51	111
AML314	D0	B3	21	2	0	9	12	1	0	37	0	2	0	0	6	0	0	0	34	0	0	0	38.0
	D31	B4	0	9	0	17	11	13	0	0	0	192	37	0	5	3	5	1	18	0	17	0	18.0
AML328	D0	B5	12	12	52	22	44	0	2	19	143	9	79	214	75	2	7	1	8	281	3	1	108
	D29	B8	15	0	13	34	82	3	25	63	365	76	6	19	134	12	5	7	16	721	0	2	282
	D113	B6	9	1	15	84	7	0	3	11	29	42	10	78	280	0	20	0	4	26	0	2	1408
	D171	B7	5	0	2	49	18	0	1	22	63	16	0	15	137	0	3	2	5	79	0	1	984
AML329	D0	B9	16	12	25	8	7	13	134	2	9	45	33	11	29	2	4	0	13	67	8	7	80
	D20	B10	25	81	74	33	4	12	39	6	10	9	336	17	30	1	1	0	19	108	51	11	86
	D37	B11	1	17	4	2	6	7	4	2	0	30	74	4	13	12	2	0	5	4	11	0	26
AML371	D0	B12	2	34	0	1	6	54	0	26	0	1	163	0	1	1	2	0	27	0	57	0	3
	D34	B13	2	15	0	7	8	20	0	7	0	30	48	0	3	8	4	1	15	0	19	0	17.0
AML419A	D0	B14	23	7	59	3	6	1	16	2	52	1	56	572	2	1	2	2	6	360	5	9	4
AML420B	D0	B15	12	1	1	10	29	11	30	7	33	70	13	2	16	0	32	1	9	34	0	0	174
	D14	B16	0	1	0	77	0	0	0	0	1	0	0	0	77	2	402	6	0	0	0	0	716
	D35	B17	1	12	3	17	5	6	0	1	0	13	408	7	9	4	47	1	1	0	36	0	172
AML475	D0	B18	17	43	198	8	1	1	15	10	18	0	1	51	9	3	0	0	7	26	0	0	15
	D29	B19	1	7	1	7	5	1	0	1	0	34	23	0	9	1	4	0	0	0	2	0	6
AML556	D0	B20	5	1	8	22	8	5	15	2	1	26	15	973	17	1	154	2	3	1	3	1064	2
	D15	B21	3	0	0	356	14	2	0	13	0	0	0	0	535	0	93	0	68	0	0	0	119
	D31	B22	15	110	1	40	63	42	0	10	1	230	415	3	32	27	240	3	42	0	155	10	12
AML707B	D0	B23	9	5	2	8	29	34	857	45	152	5	2	0	3	1	8	7	39	347	4	12	17
	D18	B25	7	5	5	103	1	2	6	13	22	0	3	0	122	1	22	0	5	28	0	0	1328
	D41	B26	2	29	4	4	12	6	4	13	2	8	146	1	24	7	0	2	7	2	12	0	102
	D97	B27	0	6	0	1	7	6	0	0	0	9	19	1	12	2	0	2	1	0	2	0	16
	D113	B24	30	48	1	45	34	17	0	1	0	55	53	2	56	6	12	25	5	1	30	1	286
AML722B	D0	B28	0	0	0	4	1	3	0	30	0	0	0	0	2	0	0	0	36	0	0	0	3
	D49	B29	0	3	0	6	3	0	0	0	0	13	0	0	4	3	0	0	1	0	1	0	39
AML870	D0	B30	3	0	2	1	2	0	56	1	96	6	0	5	5	0	0	0	3	144	0	11	10
	D14	B31	0	0	0	10	1	0	0	3	3	0	0	0	2	0	0	0	2	4	0	0	71
AML916	D0	B32	11	0	36	7	7	1	11	33	343	2	0	0	0	16	0	37	24	384	0	1	20
AML921A	D0	B33	134	16	1346	38	2	5	392	10	507	2	18	241	97	15	1	1	4	817	9	75	83
AML997	D0	B34	0	1	0	1	5	5	0	10	0	3	1	0	0	0	1	0	54	0	1	0	1
	D35	B35	0	1	0	10	2	2	0	2	0	134	12	0	6	1	2	0	1	0	4	0	10

The dataset comprises 21 cell types across 35 batches spanning 16 patients with one or multiple time points: at diagnosis (D0) and during the follow-up ($Di, i > 0$), after undergoing chemotherapy for most patients. N and T denote similar cell types corresponding to normal (N) and tumorous (T) cells, i.e., unfunctional cells induced by AML. Those cells are also referred to with the -like suffix.

Prog-like, ProMono-like, etc.) but also leading to an abundant representation of T cells (Fig I). Indeed, malignant cell types' contribution to the overall cell mixture drops after chemotherapy, leaving room for the healthy cell types (e.g., LateEry, Mono, T, NK, Plasma, etc.). Some malignant cells reappear after approximately 20 days (Prog-like, HSC-like, cDC-like, for example), indicating potential relapses.

Overall, both the patient and time point are sources of batch effects and must be accounted for. However, they also encapsulate some actual biological signals linked to the patient's genetic identity, disease stage, or response to treatment. This poses a real challenge since the technical variations to correct are intricated with meaningful biological changes that must be preserved.

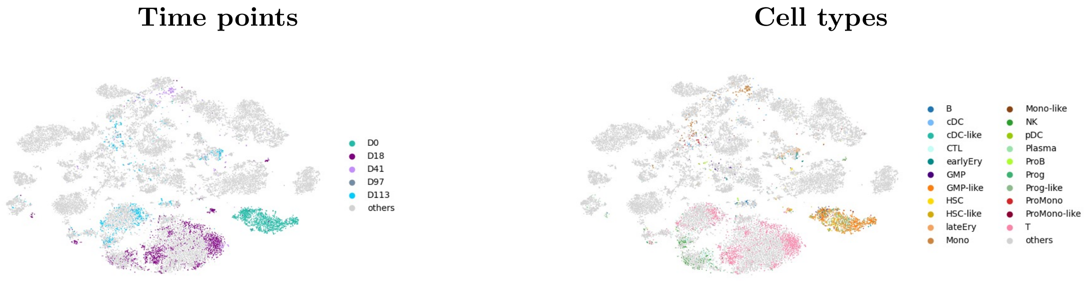

Figure H. Illustration of the time point's bias in AML707B 's cell distribution. Each patient's cell is colored by its time point (left) or cell type (right) in the t-SNE visualization.

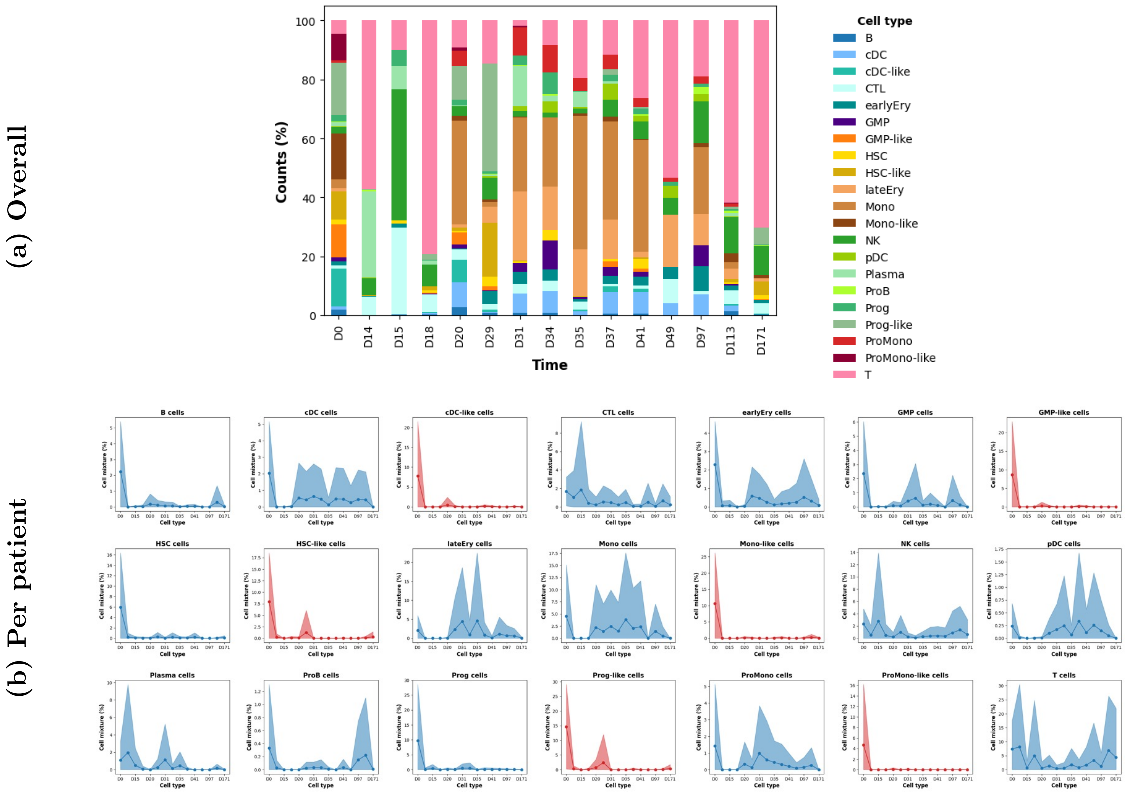

Figure I. Effect of chemotherapy: cell types' repartition over time. The evolution of the overall cell types' mixture (i.e., aggregated across all patients) is represented in the top row. The average evolution of the cell types' mixture per biopsy is depicted in the bottom row. The colors refer to the cell type's healthiness: normal (blue) and malignant (red), and the colored area corresponds to the standard deviation.

References

1. Villani AC, Satija R, Reynolds G, Sarkizova S, Shekhar K, Fletcher J, et al. Single-cell RNA-seq reveals new types of human blood dendritic cells, monocytes, and progenitors. Science. 2017;356(6335):eaah4573. doi:10.1126/science.aah4573.

2. Baron M, Veres A, Wolock SL, Faust AL, Gaujoux R, Vetere A, et al. A Single-Cell Transcriptomic Map of the Human and Mouse Pancreas Reveals Inter- and Intra-cell Population Structure. Cell Syst. 2016;3(4):346–360. doi:10.1016/j.cels.2016.08.011.

3. Muraro MJ, Dharmadhikari G, Grün D, Groen N, Dielen T, Jansen E, et al. A Single-Cell Transcriptome Atlas of the Human Pancreas. Cell Syst. 2016;3(4):385–394. doi:10.1016/j.cels.2016.09.002.

4. Segerstolpe Å, Palasantza A, Eliasson P, Andersson EM, Andréasson AC, Sun X, et al. Single-Cell Transcriptome Profiling of Human Pancreatic Islets in Health and Type 2 Diabetes. Cell Metab. 2016;24(4):593–607. doi:10.1016/j.cmet.2016.08.020.

5. Wang YJ, Schug J, Won KJ, Liu C, Naji A, Avrahami D, et al. Single-Cell Transcriptomics of the Human Endocrine Pancreas. Diabetes. 2016;65(10):3028–3038. doi:10.2337/db16-0405.

6. Xin Y, Kim J, Okamoto H, Ni M, Wei Y, Adler C, et al. RNA Sequencing of Single Human Islet Cells Reveals Type 2 Diabetes Genes. Cell Metab. 2016;24(4):608–615. doi:10.1016/j.cmet.2016.08.018.

7. Zappia L, Phipson B, Oshlack A. Splatter: simulation of single-cell RNA sequencing data. Genome Biology. 2017;18(1):174. doi:10.1186/s13059-017-1305-0.

8. Tran HTN, Ang KS, Chevrier M, Zhang X, Lee NYS, Goh M, et al. A benchmark of batch-effect correction methods for single-cell RNA sequencing data. Genome Biology. 2020;21(1):12. doi:10.1186/s13059-019-1850-9.

9. van Galen P, Hovestadt V, Wadsworth MH II, Hughes TK, Griffin GK, Battaglia S, et al. Single-Cell RNA-Seq Reveals AML Hierarchies Relevant to Disease Progression and Immunity. Cell. 2019;176(6):1265–1281. doi:10.1016/j.cell.2019.01.031.
