# Supplementary material for: A novel batch-effect correction method for scRNA-seq data based on Adversarial Information Factorization
Source: PLoS Comput Biol. 2024 Feb 22;20(2):e1011880. doi: 10.1371/journal.pcbi.1011880 (PMC10914288; doi:10.1371/journal.pcbi.1011880)
Supplement: S2 Appendix — This appendix delves into the different steps of the evaluation pipeline employed in this study to benchmark the models on the clustering and the differential expression analysis tasks. (PDF) [file pcbi.1011880.s002.pdf]

S2 Appendix: Evaluation procedure

Lily Monnier[1], Paul-Henry Cournède[1,*]
1. MICS Laboratory, CentraleSupélec, Paris-Saclay University, Gif-sur-Yvette, France.
* corresponding author: paul-henry.cournede@centralesupelec.fr

1 Clustering metrics

1.1 Adjusted Rand Index

The Adjusted Rand Index (ARI) measures the percentage of matches between two labeled lists, corrected for chance. It is based on the Rand index and ranges between 0 and 1, the latter indicating a perfect match.

$$ARI = \frac{\sum_{ij} \binom{n_{ij}}{2} - R_n}{\frac{1}{2}\left[\sum_i \binom{a_i}{2} + \sum_j \binom{b_j}{2}\right] - R_n} \quad (1)$$

With R_n being the percentage of matches corresponding to random labeling.

$$R_n = \left[\sum_i \binom{a_i}{2} \sum_j \binom{b_j}{2}\right] / \binom{n}{2} \quad (2)$$

With a_i and b_i being the number of accordant and discordant pairs in cluster i, respectively, and n_{ij} the number of common elements between the predicted cluster i and the true cluster j. Note that if the batch index is generally available, it is not usually the case for the true cell type, making this index uncomputable for unlabeled data.

1.2 Average Silhouette Width

The Average Silhouette Width (ASW) measures how similar an object is to its own cluster (cohesion) compared to other clusters (separation). The silhouette width ranges from -1 to 1, where a high value indicates that the cell is well-matched to its own cluster and poorly matched to neighboring clusters. It is defined as:

$$s(i) = \frac{b(i) - a(i)}{\max(a(i), b(i))} \text{ if } |C_i| > 1 \quad (3)$$

with $a(i)$ being the mean distance between sample i and all the other samples belonging to the same cluster, $b(i)$ being the smallest distance between sample i and all the other samples not belonging to the same group, and C_i being the i-th cluster.

1.3 Local Inverse Simpson Index

The Local Inverse Simpson Index (LISI) first defines a neighborhood based on a fixed perplexity in the local distributions and then computes the Inverse Simpson Index on this neighborhood. The Simpson Index is related to the frequency of appearance f_c of each label c within the defined neighborhood $\mathcal{N}(i)$:

$$SI(i) = \sum_{c \in \mathcal{N}(i)} f_c \quad (4)$$

This metric represents the labels' diversity within the defined neighborhood and, as such, is supposed to be close to the number of batches when applied to batch mixing and close to 1 when applied to cell type purity. In this study, we used a perplexity of 30 when computing LISI metrics.

2 Embedding and clustering algorithms

2.1 Dimensionality reduction

First, dimensionality reduction is performed, and the embedded space will then serve as the support of the metrics' computation. We preferred using t-SNE or UMAP rather than PCA as the latter often failed to capture the underlying biological information compared to the others, thus biasing any clustering metrics. Indeed, PCA relies on linear combinations of the original features, and as such, it can only capture linear effects. Furthermore, the clustering metrics are consistent across t-SNE-based and UMAP-based evaluation frameworks (S5 Appendix). The t-SNE yields better-separated clusters on the small datasets, resulting in higher ASW metrics. On the large dataset (Dataset 2), UMAP enables a finer resolution in the embedded space thanks to the higher number of components produced, better capturing the differences between cell types, thus improving the clustering results. On the AML cohort (Dataset 5), the t-SNE resulted in higher clustering results for both the original and corrected data.

2.2 K-Means

K-Means is an unsupervised clustering algorithm that minimizes the within-cluster variances (squared Euclidean distances), given a known number of clusters. This hyperparameter is user-given and is already determined for annotated datasets. For unannotated datasets, one can select the optimal value using the Akaike Information Criterion or the Bayesian Information Criterion. Although it works satisfyingly in simple clustering tasks, K-Means has limited real-world applications: the data must be linearly separable, and even in this case, the method is usually outperformed by more recent clustering algorithms (e.g., DBSCAN, Louvain, etc.). Moreover, it is highly sensitive to the initialization of the centroids. We kept this algorithm for both versions of the small datasets (Datasets 0 and 1), as it successfully clustered the cells in most cases. However, it failed to output relevant clusters compared to Louvain for Dataset 2 norm log (S5 Appendix).

2.3 Louvain

Louvain is an unsupervised clustering algorithm designed for network community detection tasks, which optimizes the modularity of the detected communities in the input graph. The modularity measures the relative density of edges inside communities with respect to edges outside communities:

$$Q = \frac{1}{w} \sum_{i,j} \left(A_{i,j} - \gamma \frac{d_i d_j}{w} \right) \delta_{c_i, c_j} \tag{5}$$

With A being the adjacency matrix, $w = 1^T A 1$ the sum of degrees, c_i the cluster of node i, d_i the degree of node i, δ the Kronecker symbol, and γ the resolution parameter.
To parse the scRNA-seq datasets into networks, we ran the k-Nearest-neighbor (kNN) algorithm in the embedded space with $k = 20$

Unlike K-Means, Louvain does not generate a partition with a predefined number of clusters. Instead, one can adjust the resolution parameter to implicitly guide the model toward a partition with the desired number of clusters: decreasing the resolution parameter leads to a more fine-grained resolution (more clusters), whereas increasing this parameter yields a more aggregated partition (fewer clusters). Using this relation, we performed a dichotomy search on this parameter's value (with 1 as the starting point and a decreasing step factor of 2 over a maximum of 20 repetitions) until convergence. At each iteration, we analyze if the previous action (increasing or decreasing the

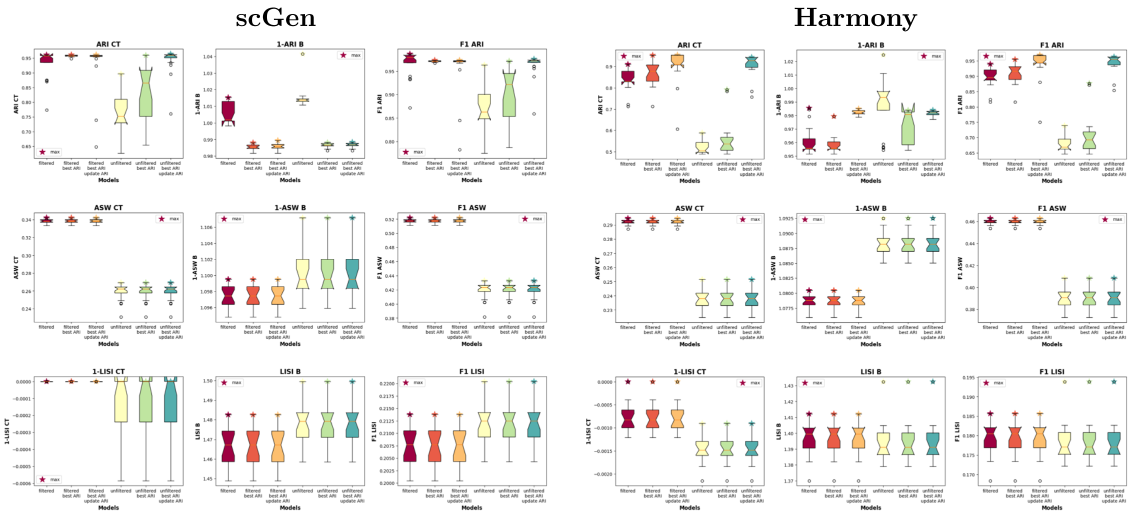

Figure A. Effects of the dichotomy search's strategies and filtering step on Louvain's clustering. Louvain is performed on the t-SNE embeddings of the batch-effect corrected data by scGen or Harmony trained on Dataset 2 norm log, with (*filtered*) a prior filtering of the scarce cell types or not (*unfiltered*). The dichotomy search is either guided by the number of clusters or the cell type ARI (*update ARI*), keeping the best clustering (*best ARI*) or not. The metrics are computed 20 times on randomly sampled 80% of the data concerning the cell type purity (CT), the batch mixing (B), or combining both criteria (F1).

resolution parameter) has improved or deteriorated the cell type ARI. In the first case, we apply the same action but with a smaller step, whereas we perform the opposite action with a smaller step in the other. When dealing with an unannotated dataset, one can use the modularity metric instead of the cell type ARI. This algorithm yielded more stable and higher and more stable results than basing the dichotomy search on the number of clusters detected compared to the actual number of clusters (Fig A).

For dataset 2, we filtered out the cells belonging to cell types having less than 50 cells in the entire dataset, i.e., t-cells (7 cells), Schwann (13 cells), MHC class II (5 cells), epsilon (28 cells), mast (32 cells). For those cell types representing less than 0.3% of the total number of cells, it is doubtful that an unsupervised method can recover their distribution. Indeed, keeping them would misguide the dichotomy search based on the number of clusters and wrongfully deteriorate the results (Fig A) by forcing the algorithm to look for more clusters than possibly retrievable. For the dichotomy search guided by the cell type ARI, we observe tighter and higher cell type ASW distributions, showing that the embedding algorithm works better when removing those noisy cells. We selected the partition corresponding to the best cell type ARI score, as the dichotomy search can stop before converging, leading to inferior clustering results (Fig A).
For the other hyperparameters, we used the default values except for $tol = 1e-4$.

3 Evaluation criteria

We computed the metrics with 20 different random seeds on the full dataset. We retained the maximum value to alleviate the bias induced by the clustering algorithm's failures, thus ensuring a fairer comparison of the model's intrinsic performances. To account for robustness, we also calculated the metrics on randomly sampled 80% of the embedded data and repeated the operation 20 times. We displayed the metrics' distribution in Appendix S5.I.

3.1 Cell type purity

We assessed cell type purity by comparing the true cell type labels with the predicted clustering labels for the ARI. We analyzed the true cell type distributions in the embedded space for the ASW and LISI metrics. We retained the maximum value over the experiments, as high cell type purity will result in high ARI, ASW, and 1-LISI scores. Note that if the batch label is generally available, it is often not the case for the cell types. To tackle this issue, one could adapt the previous definition of the cell type ASW by using the predicted K-Means labels.

3.2 Batch mixing

Only cell types shared across batches were considered for the ARI and LISI, whereas all cell types were included for the batch ASW computation, as suggested in [1]. For the ARI, the clustering was performed using the number of batches as the number of clusters. The minimum value over the experiments was retained for the ARI and ASW. In contrast, the maximum value was kept for LISI, as a superior batch mixing will result in low scores for the first metrics and a score close to the actual number of batches for LISI.

3.3 Combining cell type purity and batch mixing

To account for both criteria, we borrowed the F1-scores from [1], as defined below. Before computing the LISI F1-score, we scaled the batch and cell type metrics by subtracting the minimum theoretical value and dividing by the theoretical range to obtain values between 0 and 1.

$$F1_{ARI} = \frac{2(1 - ARI_{batch})ARI_{cell\ type}}{1 - ARI_{batch} + ARI_{cell\ type}} \tag{6}$$

$$F1_{ASW} = \frac{2(1 - ASW_{batch})ASW_{cell\ type}}{1 - ASW_{batch} + ASW_{cell\ type}} \tag{7}$$

$$F1_{LISI} = \frac{2(1 - LISI_{cell\ type})LISI_{batch}}{1 - LISI_{cell\ type} + LISI_{batch}} \tag{8}$$

4 Differential expression analysis

Before running differential expression analysis, we performed K-Means in the t-SNE subspace of the original (Raw (u)) and each model's corrected data. We used Louvain with 50 neighbors for kNN to fairly evaluate the DEGs detection performance when K-Means failed to cluster the data due to the clusters' shape (Table A). The corresponding metrics are reported in Appendix S6. All batch-effect-corrected data yield perfect clustering in terms of ARI, meaning that the DE analysis results solely reflect the models' ability to preserve the cell types' gene expression and are not impacted by the clustering algorithm's performance.

We also incorporated the results of the supervised DE analysis, i.e., using the actual cell type labels for the original data (Raw (s)). In opposition to what is presented in [1], the batch effects are not high enough to confound the supervised DEG detection, leading to high F1 scores. Thus, the supervised uncorrected DEGs' results would constitute a good sanity check for the batch-effects correction methods' preservation of the biological signal.

We investigated the model's DE performance on both the raw and log-normalized counts. On the raw counts, we clipped each cell type's genes' expression to its 0.98 and 0.02 quantiles since the test is highly sensitive to the presence of outliers (Table B). This is not the case for the log-normalized counts, showing that the outliers correspond to high total gene expression with accurate relative gene expression.

To compare the models, we computed the F1 score between the true DEGs, i.e., as intended per design in the simulation, and the predicted DEGs on the corrected data to account for both

precision and recall at a particular log-fold-change threshold. Indeed, [2] recommended filtering the DEGs called on the log-fold-change to address the test's high sensitivity. We evaluated the models at different log-fold-change thresholds to consider the performances on both lowly and highly differentially expressed genes. The results are integrated with the Area Under the Curve (AUC), and the F1 score evolutions with the log-fold-change threshold are compared.

Table A. Clustering algorithm for each model and dataset.

	Dataset 3		Dataset 3 ($n_1 = 200$)		Dataset 3 ($n_1 = 100$)		Dataset 4	
	KMeans	Louvain	KMeans	Louvain	KMeans	Louvain	KMeans	Louvain
Raw (u)	✓		✓		✓		✓	
ResPAN	✓		✓			✓	✓	
scGen		✓		✓		✓	✓	
Seurat	✓		✓		✓		✓	
AIF dyn	✓			✓		✓		✓

Table B. Sensitivity of the DE statistical test to outliers for the AIF dyn's corrected counts.

	**Log Norm**				**Raw**			
	HVG		All		HVG		All	
	Up	Down	Up	Down	Up	Down	Up	Down
Unfiltered	**0.99**	**0.91**	**0.76**	**0.71**	0.39	0	0.31	0.17
Filtered	**0.99**	**0.91**	**0.76**	**0.71**	**0.96**	**0.89**	**0.78**	**0.65**

The statistical test is performed using the unfiltered or filtered (clipping the cell type's gene expression to its 0.98 and 0.02 quantiles) raw or log-normalized counts, on the HVGs or all genes, using a log-fold-change threshold in base 2 of 0.15.

References

1. Tran HTN, Ang KS, Chevrier M, Zhang X, Lee NYS, Goh M, et al. A benchmark of batch-effect correction methods for single-cell RNA sequencing data. Genome Biology. 2020;21(1):12. doi:10.1186/s13059-019-1850-9.

2. Stuart T, Butler A, Hoffman P, Hafemeister C, Papalexi E, Mauck WM III, et al. Comprehensive Integration of Single-Cell Data. Cell. 2019;177(7):1888–1902.e21. doi:10.1016/j.cell.2019.05.031.
