# Supplementary material for: A novel batch-effect correction method for scRNA-seq data based on Adversarial Information Factorization
Source: PLoS Comput Biol. 2024 Feb 22;20(2):e1011880. doi: 10.1371/journal.pcbi.1011880 (PMC10914288; doi:10.1371/journal.pcbi.1011880)
Supplement: S3 Appendix — This appendix presents the AIF dyn architecture’s and training’s details. It also describes the training hyperparameters used for each model in the benchmark and their training time and memory usage. (PDF) [file pcbi.1011880.s003.pdf]

S3 Appendix: Training details

Lily Monnier[1], Paul-Henry Cournède[1,*]
1. MICS Laboratory, CentraleSupélec, Paris-Saclay University, Gif-sur-Yvette, France.
* corresponding author: paul-henry.cournede@centralesupelec.fr

1 AIF dyn's training details

1.1 Architecture

We borrowed scGen's VAE architecture for our CVAE: the decoder comprises two Multi-Layer Perceptron (MLP) layers of 800 units each with ReLU activations, and the encoder has two shared MLP layers composed of 800 units with ReLU activations followed by three distinct MLP layers (two for encoding for the biological signal's mean and standard deviation in the latent space and one for the batch label). The auxiliary and GAN networks are also made of two MLP layers of 800 units with ReLU activations, ensuring they will be complex enough for their respective task.

1.2 Training strategy details

For all datasets except the simulations (all versions of Dataset 3 and Dataset 4) and both versions of the counts (raw and log-normalized), we delayed the training of the auxiliary and GAN networks, updating their parameters every five epochs. The goal was to soften mainly the batch factorization constraint and, to a lesser extent, the realistic constraint, which yielded superior results in the case of batch-specific cell types and low signal-to-noise ratio (S4 Appendix). As a rule of thumb, we recommend delaying those networks' optimization for log-normalized counts since the batch effects are decreased or when dealing with batch-specific cell types because they confound the factorization constraint. For the real-world datasets' log-normalized counts and the simulated dataset's raw counts, we employed a learning rate scheduler with exponential decay of 0.999 starting after 20% of the training, which better fitted the MSE evolution, which first steeply and then slightly decreases. We would generally advise decaying the learning rate for log-normalized counts due to the corresponding MSE evolution.

1.3 Training hyperparameters

The selected AIF dyn models' training hyperparameters based on the maximum value of the F1 ARI (with reasonable F1 ASW and F1 LISI) for each dataset are detailed in Table A. Thankfully, we observe some consistency in the best hyperparameters retained across datasets' same version of the counts: $\alpha = 0.01, \beta = 0, \beta_2 \geq 0.95$ for raw counts and $\alpha = 0.05, \beta = 0.05, \beta_2 \leq 0.95$ for log-normalized counts (except Dataset 5), $\delta = 0.1$ and $\mu = 0.1$ for all. Dataset 5's lower KL weight and learning rate are justified by the log-normalization step not being the same, yielding a higher variance in the gene expression. Overall, the main differences reside in ρ and γ values. For Dataset 1, the high γ value counter-balances the softened factorization constraint. For the Dataset 0 norm log, delaying the auxiliary networks resulted in an increased variance in the auxiliary network's gradients, which is not particularly beneficial as the batch effects are minor and there are no batch-specific cell types. To counter this effect, the high ρ value sets a higher focus on the batch classification for the reconstruction.

Table A. Selected AIF dyn models' training hyperparameters

	Raw						Log norm			
	D0	D1	D3	D3 ($n_1 = 200$)	D3 ($n_1 = 100$)	D4	D0	D1	D2	D5
delayed networks	✓	✓					✓	✓	✓	✓
decay lr			✓	✓	✓	✓	✓	✓	✓	✓
epochs	1500	1500	1500	1500	1500	1500	1500	1500	1500	1500
lr	0.01	0.01	0.001	0.001	0.001	0.001	0.01	0.01	0.001	1e-5
bs	32	32	32	32	32	32	32	32	64	256
β_1	0.7	0.9	0.8	0.9	0.8	0.8	0.9	0.8	0.7	0.8
β_2	0.99	0.99	0.99	0.99	0.99	0.95	0.9	0.95	0.9	0.95
α	0.01	0.01	0.01	0.01	0.01	0.01	0.05	0.05	0.05	0.0001
β	0	0	0	0	0	0	0.05	0.05	0.05	0.05
δ	0.1	0.1	0.1	0.1	0.1	0.1	0.1	0.1	0.1	0.1
γ	0.3	0.8	0.2	0.2	0.2	0.2	0.4	0.3	0.5	0.4
μ	0.1	0.1	0.1	0.1	0.1	0.1	0.1	0.1	0.1	0.1
ρ	0.5	0.5	0.4	0.4	0.4	0.4	0.7	0.5	0.4	0.6

The following characteristics are listed: delayed training of auxiliary and GAN networks, decaying the learning rate, total number of epochs, initial learning rate (lr), batch size (bs), ADAM's coefficients (β_1, β_2), losses' weights (α for KL, β for $\hat{\mathcal{L}}_{class}$, δ for $\mathcal{L}_{gan}$, γ for $\mathcal{L}_{aux}$, μ for $\mathcal{L}_{proj}$, ρ for $\mathcal{L}_{class}$).

2 Other methods' hyperparameters

Regarding the other methods' hyperparameters, we selected the best hyperparameters inferred in the benchmark presented by [1] when possible since we used the same datasets. For scVI [2] and ResPAN [3], we employed the hyperparameters recommended by the authors as [3] incorporated some of the datasets and scVI in their analysis and both models yielded satisfying results. Since the architecture recommended by the authors (further denoted scVI (r)) has a lower complexity for the VAE part than scGen and AIF dyn, we designed another version (referred to as scVI (i)) with the same architecture as scGen and AIf dyn.

The hyperparameters used for each method are:

- **Harmony**: 30 components for the dimensionality reduction step using PCA, the iterative clustering procedure utilizes 50 clusters and is optimized for a maximum of 100 iterations with up to 20 KMeans iterations at each step, and $\lambda = 1$.
- **LIGER**: a threshold of 0.1 for defining the HVGs, an embedded space of dimension 20, 3 repetitions, and $\lambda = 5$.
- **scGen**: the same architecture as AIF dyn, a batch size of 32 for small datasets and 64 for dataset 2 norm log, a learning rate of 0.01, and the model is trained during 100 epochs.
- **scVI (r) and (i)**: a batch size of 32, a gene likelihood based on a negative binomial distribution, the model is trained during 100 epochs, and the corrected data is obtained using 20 samples.
- **ResPAN**: 20 components for the dimensionality reduction step using PCA, extracting the 2,000 HVGs between the batches on the og-normalized counts, sub-sampling the random-walks pairs generated to 3,000 pairs, a batch size of 1,024, a learning rate of 0.0001, $\lambda = 1$ and the model is trained during 300 epochs.

3 Scalability: running time and memory usage

In this section, we investigate the running time and memory usage required by each method. The running time's and memory usage's evolution with the number of cells are displayed in Fig A.

In general, all methods see their running time increasing with the dataset size, except LIGER, whose running time is nearly constant across datasets. scGen is the fastest method to perform the

batch effect correction across all human blood datasets (Datasets 0 and 1) and compares to LIGER on the largest dataset. AIF dyn requires longer training than the other deep learning methods (scGen, scVI, and ResPAN) since it does not answer the same problem. Indeed, scGen learns the cell distributions tainted by the batch effects and deconvolutes the batch effects in a supervised fashion. As such, it is a less complex problem than learning the entanglement between biological signals and technical variations in conditional distributions. ResPAN only works on a gene subset (2,000 HVGs in either of the two batches), which drastically simplifies the task ($< 8\%$ of the total genes for Datasets 0 and 1 and $< 13\%$ of the total genes for Dataset 2). Besides, it learns how to project cells onto one reference batch only, whereas AIF dyn simultaneously learns all batches' distributions. It means that in practice, we need to re-run ResPAN to obtain the projection onto another reference batch, which can be the case when using different technologies (SMART-seq2 and 10xgenomics for example) or modalities (scRNA-seq and sc-ATAQ, for example). Similarly to scGen, scVI learns the cells' distribution with the batch effects. Indeed, it does not enforce any factorization of the technical variations in the latent space, resulting in poorer batch mixing results. Moreover, AIF dyn's more complex formulation allows a better preservation of the biological signal in the DEG analysis than scGen and ResPAN, thanks to the projection constraint and the MSE in the encoder's objective.

AIF dyn is the best model for memory usage thanks to its stochastic optimization relying on mini-batch gradient descent and its batch-effect correction method, which can be performed by batch.

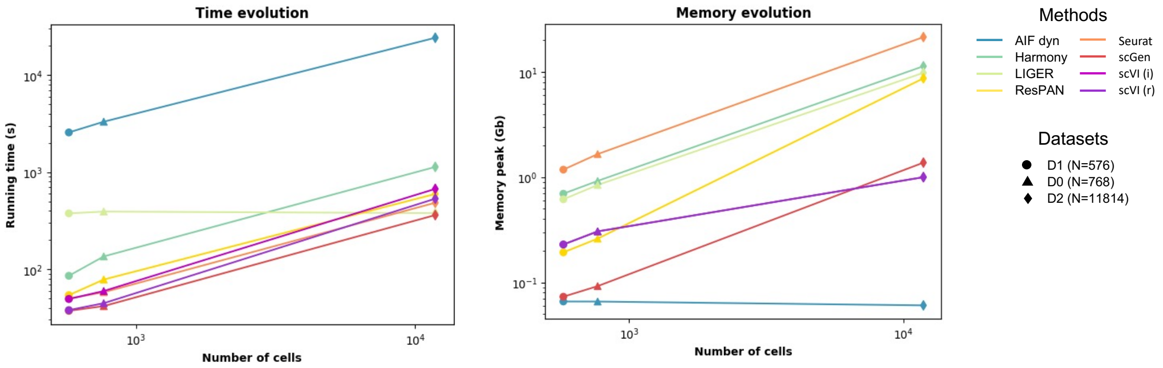

Figure A. Running time and memory usage. Comparison of the evolution of each method's running time and memory usage with the number of cells.

On a side note, we believe the training time could be significantly decreased by better-suited losses' normalization and weighting strategies since all losses are quickly stagnating, except the KL and the factorization constraint for Dataset 2 norm log (Fig B). The clustering results are already quite good after 300 and 500 epochs for Dataset 0 norm log and Dataset 2 norm log, respectively. Indeed, AIF dyn's performance after 300 epochs reaches scGen's, and the gain of the last 1,200 epochs is relatively small ($< 3\%$ for the F1 ARI and F1 LISI) and mainly affects the ASW metrics for Dataset 0 norm log. For the Dataset 2 norm log, the last 1,000 epochs focus on improving batch mixing while maintaining the same quality of cell type purity. We observed in some cases that the cell type purity and the batch mixing can be competing objectives, especially in the case of batch-specific cell types, leading to a cycling optimization of the two tasks successively, manifested by an improvement of the currently optimized task while deteriorating the other. Thus, we believe a better optimization (losses' normalization and losses' weights definition) of the encoder's overall objective could significantly shorten the training time.

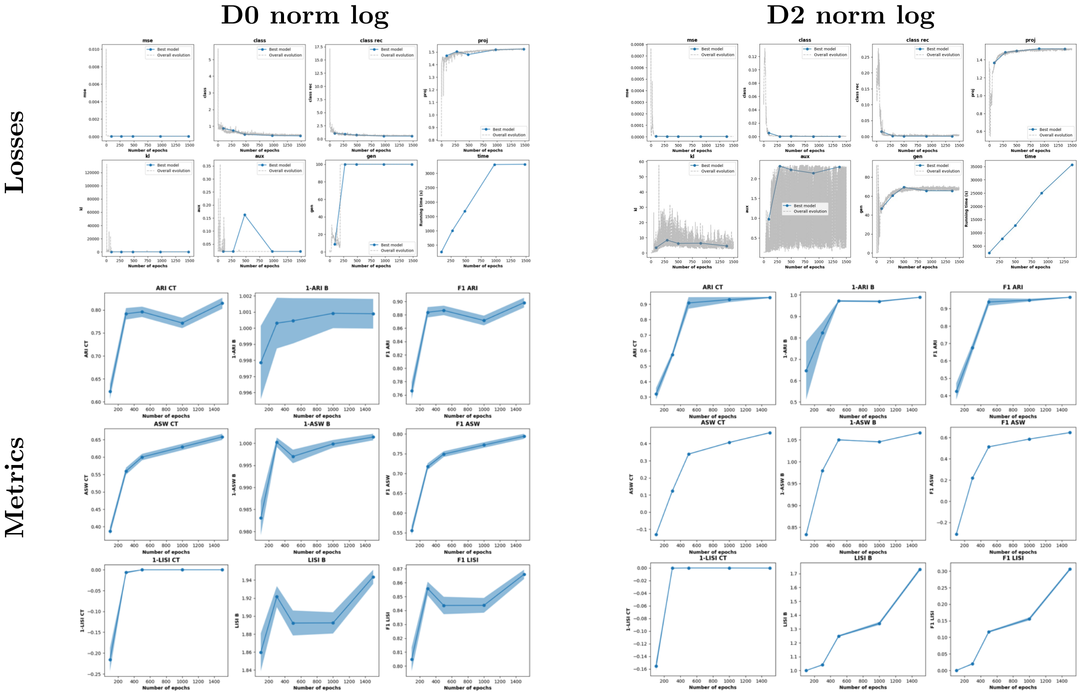

Figure B. AIF dyn's performance evolution. Evolution of AIF dyn's losses and clustering metrics during training. The metrics are computed on 80% on the corrected data using K-Means on the t-SNE embeddings for Dataset 0 norm log and Louvain on the UMAP embeddings for Dataset 2 norm log, concerning the cell type purity (CT), the batch mixing (B) or combining both criteria. The metrics' mean (line) and standard deviation (area) are reported. The losses' overall evolution during training, along with the best models at 100, 300, 500, 1,000, and 1,500 epochs, are presented.

References

1. Tran HTN, Ang KS, Chevrier M, Zhang X, Lee NYS, Goh M, et al. A benchmark of batch-effect correction methods for single-cell RNA sequencing data. Genome Biology. 2020;21(1):12. doi:10.1186/s13059-019-1850-9.

2. Lopez R, Regier J, Cole MB, Jordan MI, Yosef N. Deep generative modeling for single-cell transcriptomics. Nat Methods. 2018;15(12):1053–1058. doi:10.1038/s41592-018-0229-2.

3. Wang Y, Liu T, Zhao H. ResPAN: a powerful batch correction model for scRNA-seq data through residual adversarial networks. Bioinformatics. 2022;38(16):3942–3949. doi:10.1093/bioinformatics/btac427.
