# Supplementary material for: A novel batch-effect correction method for scRNA-seq data based on Adversarial Information Factorization
Source: PLoS Comput Biol. 2024 Feb 22;20(2):e1011880. doi: 10.1371/journal.pcbi.1011880 (PMC10914288; doi:10.1371/journal.pcbi.1011880)
Supplement: S4 Appendix — This appendix summarizes the AIF dyn’s main results regarding the robustness of the multi-sample batch-effect correction method, the influence of the losses’ weights, the benefits of the dynamic ratio, the projection constraint, and delaying the auxiliary and GAN networks. We also investigated the model’s ability to generalize to unseen samples compared to scGen. (PDF) [file pcbi.1011880.s004.pdf]

S4 Appendix: AIF dyn's main results

Lily Monnier[1], Paul-Henry Cournède[1,*]
1. MICS Laboratory, CentraleSupélec, Paris-Saclay University, Gif-sur-Yvette, France.
* corresponding author: paul-henry.cournede@centralesupelec.fr

1 A robust batch effect correction method

To assess the benefits of the stochastic batch effect correction method (generating several samples from the latent distribution) compared to the deterministic one (using the estimated mean as the latent vector), we implemented both strategies on the same AIF model trained on Dataset 1. We compared their clustering metrics (Table A) using K-Means on PCA embeddings. The deterministic method yields surprisingly low results in matching the true labels (ARI cell type) and the quality of the clusters' structure (ASW cell type), thus resulting in low F1 ARI and F1 ASW scores. It highlights that the mean does not represent the latent distribution well enough. Indeed, the deterministic strategy does not benefit from the variational aspect of the CVAE: it does not use the full information embedded in the latent representation distribution but solely the mean.

Table A. Comparison of the batch effect correction strategies on Dataset 1.

	ARI			ASW		
	CT	1-B	F1	CT	1-B	F1
deterministic	0.26	1.00	0.41	0.16	0.95	0.27
s(1)	0.74	1.00	0.85	0.31	0.97	0.47
s(20)	0.80	1.00	0.90	0.56	0.95	0.70

The metrics are computed on cell type purity (CT), batch mixing (B), and combining both criteria (F1).

To assess the robustness of multiple sampling (s(20) for 20 samples drawn from the latent distribution) against the unique sampling strategy (s(1)), we analyzed the clustering metrics variability when repeating the batch-effect correction step with different random seeds on the same AIF model trained on Dataset 1. The results are summarized in Table B. We observe that not only are all metrics significantly improved in terms of average when using multiple sampling but also the metrics' variability is decreased by a factor of 10. Thus, it counters stochasticity's main drawbacks by enhancing the strategy's robustness.

Table B. Effect of the multiple sampling strategies on the model's robustness.

	ARI			ASW		
	CT	1-B	F1	CT	1-B	F1
s(1)	0.68 ± 0.06	1.00	0.81 ± 0.04	0.32 ± 0.01	0.97	0.48 ± 0.01
s(20)	0.83	1.00	0.91	0.56	0.95	0.70

The mean and standard deviation are computed over 20 repetitions of batch effect correction on Dataset 1. The metrics are computed on cell type purity (CT), batch mixing (B), and combining both criteria (F1).

We also investigated the effect of the batch label used for projection for datasets 0 and 1. We observed no significant change, indicating that, for those use cases, no optimization is required for this hyperparameter.

2 Influence of the losses' weights

In this section, we investigated the influence of the losses' weights defined in Eq 1 on the AIF model's performance on the raw versions of Dataset 0 and Dataset 1:

$$\begin{cases} \mathcal{L}_{enc} = & \mathcal{L}_{rec} + \alpha\mathcal{L}_{KL} + \rho\mathcal{L}_{class} + \beta\hat{\mathcal{L}}_{class} - \mu\mathcal{L}_{proj} - \delta\mathcal{L}_{gan} - \gamma\mathcal{L}_{aux} \\ \mathcal{L}_{dec} = & \mathcal{L}_{rec} + \beta\hat{\mathcal{L}}_{class} - \delta\mathcal{L}_{gan} - \mu\mathcal{L}_{proj} \end{cases} \tag{1}$$

with $\mathcal{L}_{rec} = MSE(\hat{x}, x)$, $\mathcal{L}_{KL} = KL\left[q_\phi\left(z|x\right)||p(z)\right]$, $\mathcal{L}_{class} = \mathcal{L}_{ce}(\hat{y}, y)$, $\hat{\mathcal{L}}_{class} = \mathcal{L}_{ce}(\hat{\hat{y}}, y)$, $\mathcal{L}_{aux} = \mathcal{L}_{ce}(\tilde{y}, y)$, $\mathcal{L}_{proj} = \mathcal{L}_{cos}(\hat{x}, \hat{x}_{avg}) + \mathcal{L}_{cos}(\hat{x}, \hat{x}_{rand})$,
$\mathcal{L}_{gan} = \frac{1}{3}(\mathcal{L}_{ce}\left(y_{real}, C_\chi\left(x\right)\right) + \mathcal{L}_{ce}\left(y_{fake}, C_\chi\left(D_\theta\left(E_\phi\left(x\right)\right)\right)\right) + \mathcal{L}_{ce}\left(y_{fake}, C_\chi\left(D_\theta\left(z\right)\right)\right))$.

We trained the models several times (10 times), changing the random seed to account for the convergence's variability. Although the KL term has a twofold purpose: regularizing the latent space distribution and tightening the gap between the ELBO and the true objective, [1] highlighted that a higher value deteriorated the results by relatively decreasing the other objectives (e.g., reconstruction, classification). Thus, we mainly focused on the roles of the auxiliary, the GAN, and the classification losses in the overall encoder's loss, and explored the following values: $\alpha = 0.2$, $\rho \in \{0.1, 0.5, 1\}$, $\beta \in \{0, 0.1\}$, $\gamma \in \{0, 0.1, 0.5, 1\}$, $\delta \in \{0, 0.1, 0.5, 1\}$, $\mu = 0$.

We trained each model with different values of β_1 (0.7, 0.8 or 0.9) and β_2 (0.9, 0.95 or 0.99) to alleviate the bias induced by ADAM's coefficients, directly impacting the model's optimization process. We aggregated the final losses using the median over the experiments (10 repetitions) and the training hyperparameters (all β_1 and β_2 values tried). We also computed the clustering metrics using K-Means on the PCA embeddings of the corrected data to account for the model's ability to accurately correct the batch effect while preserving the underlying biological signal. We aggregated them similarly to the training losses. The results are shown in Fig A for Dataset 0 (left column) and Dataset 1 (right column).

2.1 Benefits of the factorization constraint

In Fig A, the factorization constraint, whose strength is embedded by γ, enhanced the reconstruction objective for both datasets, especially under a low focus on the classification-derived objectives. Thus, forcing the optimal state to be factorized helped the model converge. Indeed, its contribution is two-fold. By depriving the latent vector of the batch information, the model is incited to: 1) capture more batch information in the predicted batch label, improving the classification objective, 2) purify the biological signal, refining the estimation of the latent representation distribution. This assumption is confirmed by higher cell type ARI, 1 - batch ARI, and cell type ASW metrics since the batch-effect correction step relies on the quality of both the estimated batch label and the batches' cell distributions inferred. This proves the interest in adapting Adversarial Information Factorization to batch-effect correction.

2.2 Benefits of the distribution-based reconstruction objective

The distribution-based reconstruction objective generated by the GAN block, whose importance is controlled by δ, is particularly beneficial for the model's reconstruction convergence under low classification focus and factorization constraint for both datasets. By enforcing the model to generate realistic samples, the pairwise reconstruction constraint, embedded by the MSE, is softened into a distribution-based reconstruction constraint, thus allowing samples far from the original samples but within one cell type distribution. Although this could be both favorable and unfavorable to the optimization process, depending on the cell type distribution the samples lie in, we observe that it mainly improves the model's reconstruction performances, resulting in higher ARI and ASW metrics for Dataset 1. However, it does not translate in terms of clustering metrics for Dataset 0, which could be due to the presence of reconstructed cells outside of their cell type distribution, leading to a deterioration of the clustering performance. The higher benefits of the distribution-based

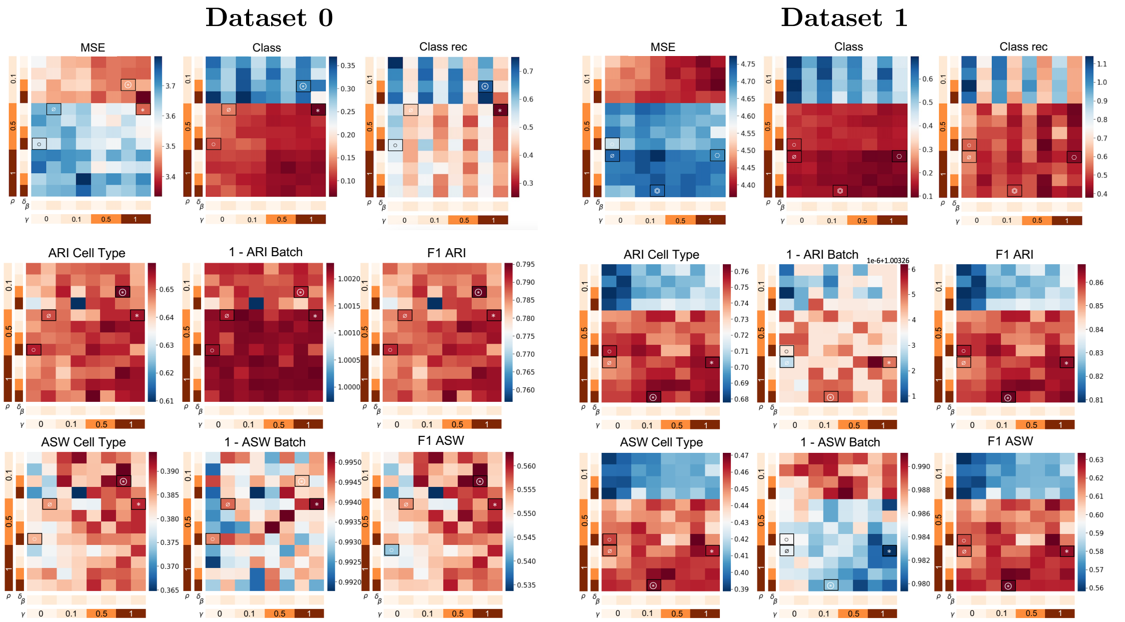

Figure A. Influence of the losses' weights on the AIF model's convergence and batch-effect correction performances. The reconstruction and classification training losses (first row) and the cell type and batch clustering metrics (second and last rows) for Dataset 0 (left column) and Dataset 1 (right column) are represented. The best model in terms of F1 ARI in each category is also outlined for each dataset: CVAE (∅), CVAE + AUX (∗), CVAE + GAN (◦), CVAE + AUX + GAN (AIF, ⊛).

reconstruction objective for Dataset 1 can be explained by the fewer samples and the batch-specific cell types, thus requiring exploiting the information embedded in the cell distributions.

2.3 Importance of the classification objectives

As expected, the classification-derived losses are improved when increasing the focus on the classification objective in the overall encoder's loss. However, augmenting the classification weight ρ led to a deterioration of the MSE for both datasets, which relatively decreased its importance. Regarding clustering metrics, it resulted in higher cell type and 1 - batch ARI for both use cases, indicating that the batch label estimation is crucial for the batch-effect correction step: it relies on it to project one batch's cell distribution onto the other's one. Nevertheless, the ASW metrics decline when the classification weight ρ increases for Dataset 0, which is directly related to the deterioration of the MSE, as the ASW is based on distances between cells. Although the 1 - batch ASW declines for Dataset 1, the cell type ASW is enhanced, improving the F1 ASW, thus highlighting the classification objective's greater importance in batch-specific cell types.

One might consider using a classification weight greater than 0.1, as the model struggles to optimize the classification loss: it starts re-increasing after about 300 epochs, as observed in Fig B. However, a low classification weight yields better-converged results regarding the MSE evolution: when models with high classification weights start stagnating, those with low classification weights still decrease with a relatively steep slope and lie lower than the others. Hence, one should further train the models with low classification weights to determine the optimal ρ value.

The classification after reconstruction loss is improved when incorporating this term into the encoder's loss. Moreover, the classification loss decreases when adding the classification-after-reconstruction term, showing that the model benefited from the reconstructed samples' features in the classification optimization process. It can be explained by the model being trained upon more samples (original and reconstructed), thus better adapting its parameters towards generalization.

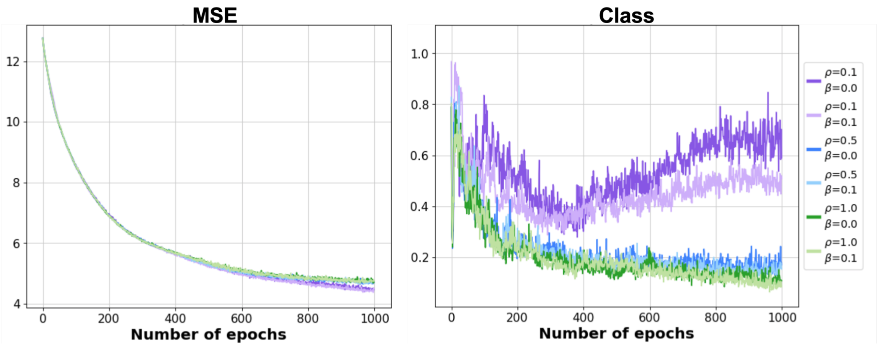

Figure B. Losses' weights influence on the AIF training losses evolution. The results correspond to an AIF model with $\alpha = 0.2, \gamma = 0.1, \delta = 0.1, \mu = 0$ trained on Dataset 1.

The reconstruction objective evaluated through the MSE is enhanced when adding the classification-after-reconstruction term for both datasets, especially for high factorization constraints, as it embeds a constraint on retaining the batch information in the reconstructed samples. However, it does not translate regarding clustering metrics, as no particular trend is observed.

The (1-ASW) batch metric is deteriorated by the factorization constraint and the classification weight for both datasets, which appears contradictory. Indeed, the factorization constraint and the classification weight should improve the batch effect correction regarding the batch mixing abilities, as they refined the batch label estimation. However, the deterioration of the metric could result from a better reconstruction of the unshared cell types as the batch ASW is computed on all cell types.

2.4 Ablation study

In Fig A, we outlined the best model in terms of F1 ARI for each category: a classic CVAE ($\varnothing$), the CVAE with the factorization constraint (CVAE + AUX, represented by $*$), the CVAE combined with the GAN (CVAE + GAN, symbolized by $\circ$), and the AIF (CVAE + AUX + GAN, corresponding to $\circledast$) formulation composed of the CVAE, the factorization constraint and the GAN objective. First, the factorization constraint yields an equivalent batch mixing for an enhanced cell type purity for the low signal-to-noise ratio setting (dataset 0). It improves both the CVAE's batch mixing and cell type purity performances in the most complicated setting, where some cells were batch-specific (dataset 1). Second, the distribution-based reconstruction objective induced by the GAN block enhances the CVAE's cell type purity ability for both datasets compared to its classic formulation. Finally, the combination of the two additional blocks, corresponding to the AIF model, surpasses all other formulations in terms of F1 ARI and F1 ASW metrics on both datasets, showing the joint benefits of those blocks. Thus, both the GAN and auxiliary blocks are proved to be necessary.

2.5 Conclusion

The factorization and the realistic constraints helped improve the AIF model's performance by guiding its convergence for both datasets, resulting in higher cell type purity and batch mixing performances. However, a tradeoff appears between the different components of the encoder's loss, and the optimal values should be inferred as they depend on the use case considered. We did not observe any explicit linear correlation between the training losses (MSE, classification, and classification after reconstruction) and the clustering metrics aggregated over the experiments that were validated across the weights' combinations. This is due to the clustering task's bias in the metrics, favoring models ensuring that the reconstructed samples cluster within their cell types, regardless of the potential alteration of the biological signal, which is best evaluated through the MSE.

3 Benefits of the dynamic ratio

This section investigates the benefits of using a small dynamic ratio during the losses' normalization step. In Fig C, we represented the losses' evolution for the AIF rel and AIF dyn models trained on the raw version of Dataset 2, where the latter relies on a more focal optimization process as the median is computed using only the last 10% of the total number of epochs instead of all the previous epochs. We chose the raw version: as it is more unstable than the pre-processed one, it will better illustrate the dynamic ratio's benefits in the case of arduous training. Having a more focal optimization process is supposed to give higher importance to wrongly optimized losses in the recent steps, allowing the network to counter the misleading steps more quickly and efficiently, which is what we observe for the MSE, the KL, and the classification derived losses in Fig C. Indeed, although the peaks are higher, they are tighter for AIF dyn than for AIF rel, proving that the dynamic version was quicker and more efficient in correcting the misleading steps.

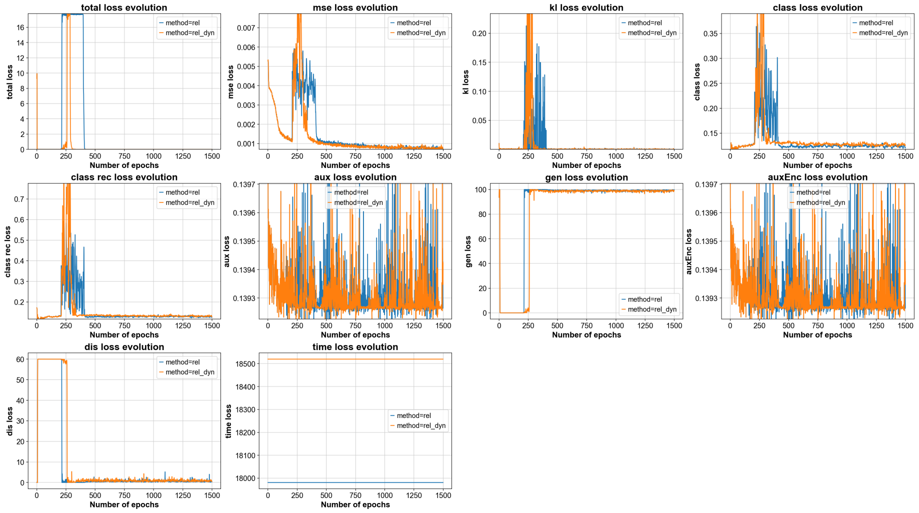

Figure C. Influence of the dynamic ratio on the losses' evolution. The results correspond to the AIF rel (blue) and AIF dyn (orange) models ($\alpha = 0.2$, $\gamma = 1.0$, $\delta = 0.1$, $\rho = 0.5$, $\beta = 0.1$, $\mu = 0$) trained on the raw version of Dataset 2 with a batch size of 64, a learning rate of 0.001, $\beta_1 = 0.9$ and $\beta_2 = 0.99$.

4 Benefits of the projection constraint

The projection constraint $\mathcal{L}_{proj}$ was incorporated to provide some regularization in the latent space and cross-over between each batch's cell distribution. Indeed, it forces the decoder to output similar reconstructions (in terms of cosine similarity) for the same latent vector z across batches while keeping some differences inherent to the batch effects, contrarily to an MSE, which is desired at this step as we aim to learn the batch-conditional cell types' distribution. Thus, it enhances the quality of the batch-effect correction step which relies on cells' projection, improving the DE analysis results and removing negative gene expression values. Indeed, in Table C, the benefits of the ReLU post-processing of the corrected data observed for the AIF dyn without projection constraint disappear when adding this constraint to the overall CVAE's objective. Moreover, it allows the decoder to see all cell types for each batch, thus addressing the distribution's singularity posed by batch-specific cell types.

Table C. Influence of the projection constraint on the AIF dyn's DEG results.

	**Raw**				**With ReLU**			
	HVG		All		HVG		All	
	Up	Down	Up	Down	Up	Down	Up	Down
w/o proj	0.94	0.66	0.63	0.42	0.97	0.67	0.65	0.50
with proj	**0.99**	**0.91**	**0.76**	**0.71**	**0.99**	**0.91**	**0.76**	**0.71**

The comparison is based on the up and down-regulated DEGs F1 score evaluated on the simulated Dataset 3 log-normalized counts, with or without ReLU post-processing and a log-fold-change threshold of 0.15 in base 2.

5 Benefits of delaying the auxiliary's and GAN's training

We delayed the training of the auxiliary network and GAN, updating their parameters every 5 epochs, to soften mainly the batch factorization constraint and to a lesser extent the realistic constraint. In Table D, we compared the best AIF dyn models with normal training (AIF dyn (N)) and delayed training (AIF dyn (D)) on Dataset 0 and Dataset 2 norm log. The cell type's and batch's ARI and LISI metrics are improved when the batch factorization and realistic constraints are softened, indicating higher cell type preservation and batch-mixing abilities. In the case of a low signal-to-noise ratio (Dataset 0), the batch effects are higher than the biological signal. Thus, softening the batch factorization constraint helps the model focus on preserving the biological signal. It also benefits batch-specific cell types (Dataset 2 norm log) by less penalizing their existence.

Table D. Comparison of the AIF dyn's normal (N) and delayed training (D).

	**Dataset 0**									**Dataset 2 norm log**								
	ARI			ASW			LISI			ARI			ASW			LISI		
	CT	1-B	F1	CT	1-B	F1	CT	B	F1	CT	1-B	F1	CT	1-B	F1	CT	B	F1
AIF dyn (N)	0.82	1.00	0.90	0.53	1.00	0.69	1.01	1.89	0.84	0.92	0.97	0.94	0.26	1.07	**0.42**	1.00	1.33	0.15
AIF dyn (D)	0.83	1.00	**0.91**	0.59	1.00	**0.74**	1.02	1.91	**0.85**	0.95	0.99	**0.97**	0.25	1.05	0.40	1.00	1.62	**0.27**

The comparison is based on the clustering metrics evaluated on the corrected data's t-SNE embeddings with KMeans for Dataset 0 and Louvain with a dichotomy search on the number of clusters for Dataset 2 norm log. Each metric is computed for the cell type purity (CT), the batch mixing (B), and combining both criteria (F1).

6 Ability to generalize to unseen samples

To account for robustness, we investigated the performance of scGen and the best AIF dyn model to accurately correct batch-effect for unseen samples. To this end, we compared both models' performance on the training and full datasets, composed of the training and the test sets. The results are stored in Table E. We observe a slight decrease in the ASW and LISI metrics for both models but nothing significant. Moreover, it might have resulted from a poorer performance of UMAP. Overall, both models efficiently corrected the batch effects for the samples in the test set, showing that the models did not overfit and could generalize to unseen samples.

Table E. Comparison of the methods' generalization ability to unseen samples.

	**Train set**									**Train and test sets**								
	ARI			ASW			LISI			ARI			ASW			LISI		
	CT	1-B	F1	CT	1-B	F1	CT	B	F1	CT	1-B	F1	CT	1-B	F1	CT	B	F1
scGen	0.96	0.99	**0.98**	0.55	1.06	**0.72**	1.00	1.49	0.22	0.96	0.99	**0.97**	0.55	1.07	**0.73**	1.00	1.46	0.20
AIF dyn	0.95	0.99	0.97	0.46	1.06	0.65	1.00	1.68	**0.29**	0.94	0.99	**0.97**	0.45	1.06	0.63	1.00	1.60	**0.26**

The metrics are calculated on the UMAP embeddings of the full corrected data on either the training set or training and test sets of Dataset 2 norm log. Each metric is computed for the cell type purity (CT), the batch mixing (B), and combining both criteria (F1).

References

1. Creswell A, Mohamied Y, Sengupta B, Bharath AA. Adversarial Information Factorization. arXiv e-prints. 2017; p. arXiv:1711.05175.
