# Supplementary material for: A novel batch-effect correction method for scRNA-seq data based on Adversarial Information Factorization
Source: PLoS Comput Biol. 2024 Feb 22;20(2):e1011880. doi: 10.1371/journal.pcbi.1011880 (PMC10914288; doi:10.1371/journal.pcbi.1011880)
Supplement: S5 Appendix — This appendix investigates the models’ clustering robustness and the biases induced in the metrics by the embedding and clustering algorithms’ performance. (PDF) [file pcbi.1011880.s005.pdf]

S5 Appendix: Models' clustering robustness and metrics' biases

Lily Monnier[1], Paul-Henry Cournède[1,*]
1. MICS Laboratory, CentraleSupélec, Paris-Saclay University, Gif-sur-Yvette, France.
* corresponding author: paul-henry.cournede@centralesupelec.fr

This section investigates the models' clustering robustness and the metrics' biases due to either the embedding method or the clustering algorithm. Indeed, the clustering metrics strongly depend on the performance of the clustering and dimensionality reduction algorithms. Although some models yielded satisfying clusterings (each cell type corresponded to a cluster) on the t-SNE or UMAP visualizations, the metrics did not reveal any improvement or even suggested a performance deterioration, underlining the bias in the metrics.

1 Models' clustering robustness

To account for the models' clustering performance robustness, we ran the clustering evaluation pipeline on randomly sampled 80% of the data and repeated the operation 20 times for each dataset. The results are displayed in Fig A. We observe low-standard-deviation distributions for all datasets, yielding the same ranking of the models as the one obtained with the metrics' maximum on the full dataset. Thus, the methods' clustering robustness strongly corroborates the superiority of AIF dyn on the human blood datasets (Datasets 0 and 1). For the human pancreas dataset (Dataset 2 norm log), the cell type ARI is more variable, comprising some outliers (LIGER, ResPAN, Seurat, and scVI (i)), which is a direct consequence of the Louvain clustering algorithm's failures. Since we do not impose any constraint on the sampling process, it is highly likely that some cell types are not represented or have very few representatives, thus hindering the clustering. This assumption is further validated by the higher and more stable F1 ARI when using the complete dataset for LIGER, ResPAN, and Seurat (Fig B).

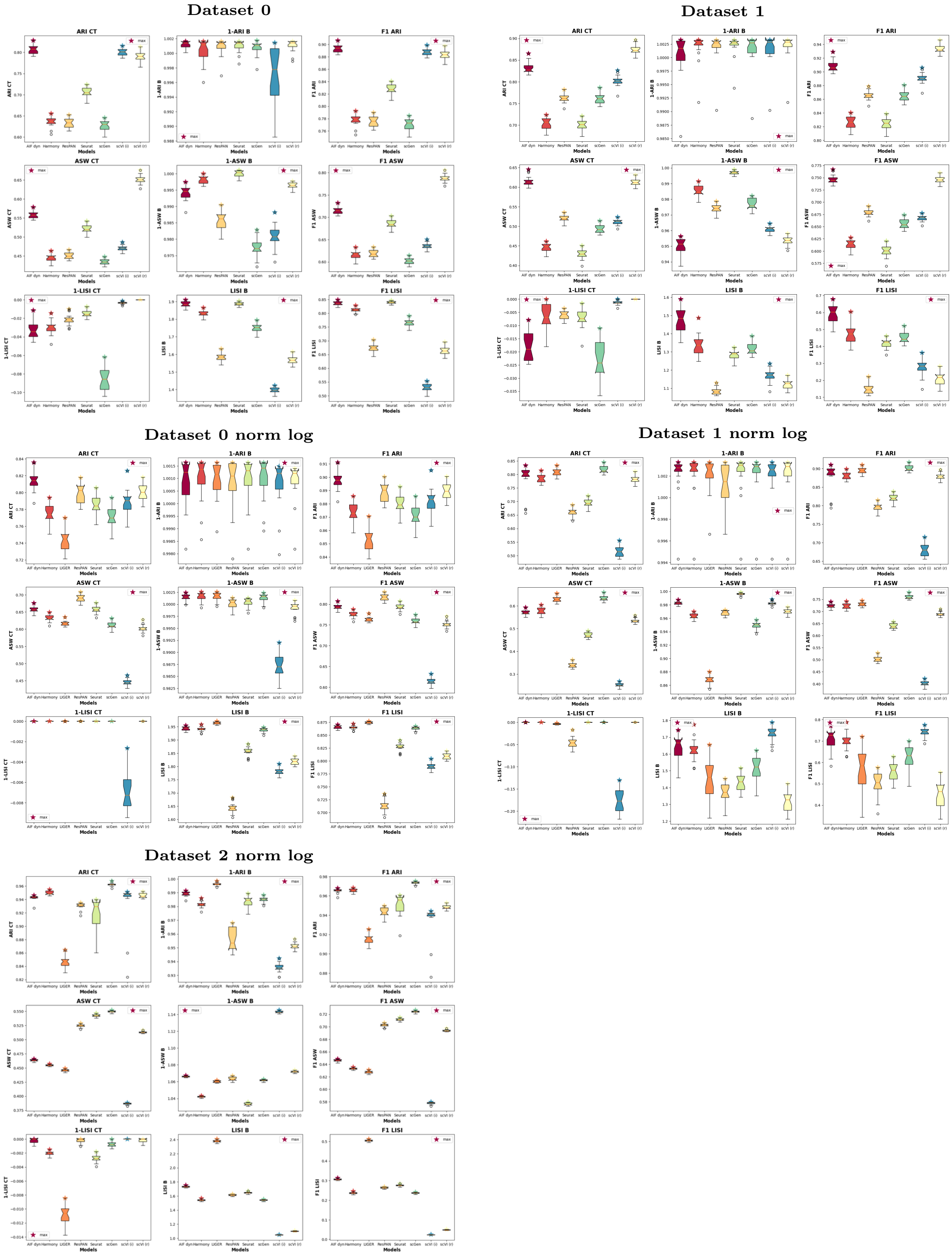

Figure A. Comparison of the methods' clustering robustness by computing the clustering metrics on 80% of the datasets. The metrics are calculated 20 times on randomly sampled 80% of the corrected data's t-SNE (Datasets 0 and 1) or UMAP embeddings (Dataset 2) for the raw and log-normalized counts. Each metric is computed for the cell type purity (CT), the batch mixing (B), and combining both criteria (F1).

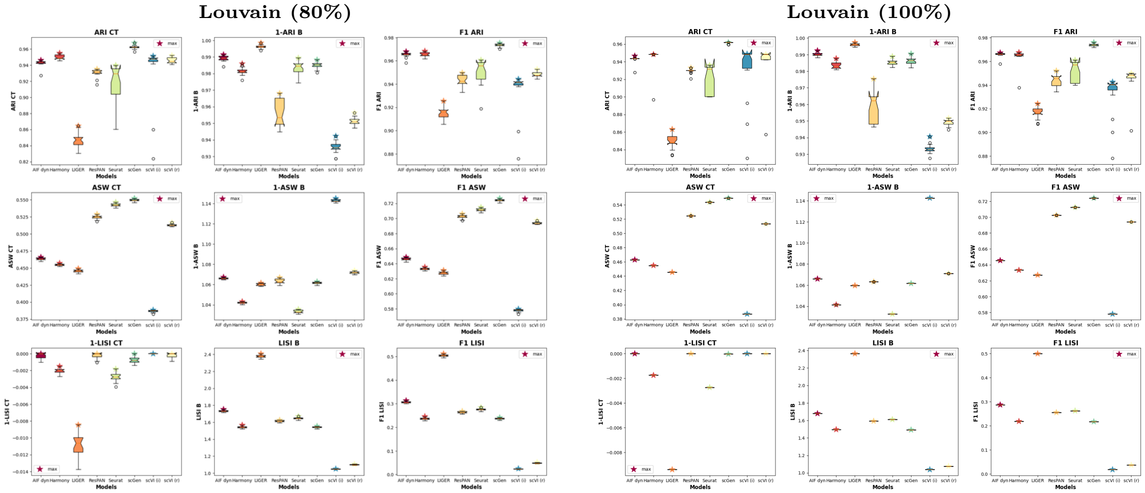

Figure B. Methods' clustering robustness using Louvain algorithm on 80% or 100% of the corrected Dataset 2 norm log. The metrics are calculated 20 times on randomly sampled 80% or 100% of the corrected data's UMAP embeddings for dataset 2's log-normalized counts. Each metric is computed for the cell type purity (CT), the batch mixing (B), and combining both criteria (F1).

2 Embedding algorithm's biases

To illustrate the bias in the metrics induced by the embedding algorithms, we reported the clustering metrics computed on each embedded space in Table A for the models presented in the comparison to the state-of-the-art section trained on either the raw or pre-processed versions of the full Datasets 0, 1, and 2. We used 20 components for PCA and UMAP and only 2 for t-SNE: although the Barnes-Hut t-SNE implementation speeds up the computation, it is limited to 3 or fewer components. We observe that, for all datasets and all methods, the metrics are highly variable from one embedding method to another, showing significant biases in the metrics and questioning their fair evaluation of the models.

For the small datasets, the t-SNE or UMAP embeddings yield better metrics. This can be explained by the PCA relying on linear combinations of the feature to produce embeddings, which often fails to capture most of the information in complex real-world datasets where the linearity assumption becomes untrue. The metrics are usually similar between those two dimensionality reduction algorithms, except for the ASW, which is usually higher for the t-SNE as the clusters are often tighter than for the UMAP. Given those observations, we recommend using the t-SNE embeddings in the metrics' computation.

For the large dataset, although the PCA fails to capture non-linear relationships between features, explaining the deterioration of the ASW and LISI metrics since the distances are altered where the data are non-linearly explainable. The lower clustering results on the t-SNE and UMAP for ResPAN are explained by a subdivision of the alpha cells, corresponding to batch-related differences. Unlike the small datasets, using UMAP embeddings produced better-structured clusters than t-SNE, resulting in a higher cell-type ASW. This is due to the higher number of components used, capturing more subtle differences between cell types. The models' ranking changes, showing that AIF dyn's lower cell type ASW in the t-SNE was due to the embedding algorithm's performance. Besides, it leads to slightly higher cell-type ARI metrics for all models except ResPAN. The batch LISI is also improved for over half of the models (scGen, Harmony, scVI (r), and AIF dyn). The UMAP-based metrics yield satisfying results for all models, corroborating the performance observed on the visualizations.

Table A. Influence of the embedding algorithm on the clustering metrics.

(a) Dataset 0

		PCA							t-SNE							UMAP						
		sG	H	Se	R	Sr	Si	A	sG	H	Se	R	Sr	Si	A	sG	H	Se	R	Sr	Si	A
ARI	CT	0.55	0.61	0.59	0.33	0.83	0.80	0.57	0.63	0.64	0.70	0.65	0.79	0.81	0.81	0.68	0.56	0.69	0.63	0.82	0.61	0.81
	1-B	0.99	1.00	1.00	1.00	1.01	1.00	1.00	1.00	1.00	1.00	1.00	1.00	1.00	1.00	1.00	1.00	1.00	1.00	1.00	1.00	1.00
	F1	0.71	0.75	0.74	0.49	**0.91**	0.89	0.73	0.77	0.78	0.83	0.78	0.88	**0.89**	**0.89**	0.81	0.73	0.82	0.78	**0.90**	0.75	0.89
ASW	CT	0.41	0.26	0.30	0.29	0.22	0.11	0.19	0.44	0.45	0.52	0.45	0.65	0.47	0.56	0.37	0.40	0.47	0.45	0.64	0.46	0.50
	1-B	0.99	1.00	0.99	0.97	0.97	0.95	1.04	0.98	0.99	1.00	0.99	1.00	0.98	0.99	0.98	1.00	1.00	0.98	0.99	0.97	0.99
	F1	**0.58**	0.41	0.46	0.45	0.36	0.19	0.33	0.61	0.62	0.69	0.62	**0.79**	0.64	0.72	0.54	0.57	0.64	0.62	**0.78**	0.62	0.66
LISI	CT	1.05	1.03	1.01	1.02	1.00	1.17	1.01	1.06	1.02	1.01	1.01	1.00	1.00	1.02	1.06	1.05	1.01	1.02	1.00	1.00	1.02
	B	1.73	1.77	1.69	1.51	1.61	1.49	1.87	1.75	1.81	1.88	1.54	1.52	1.35	1.88	1.82	1.85	1.86	1.52	1.43	1.24	1.89
	F1	0.76	0.78	0.74	0.62	0.69	0.60	**0.83**	0.76	0.80	**0.84**	0.65	0.63	0.48	**0.84**	0.77	0.82	0.83	0.63	0.56	0.37	**0.84**

(b) Dataset 0 norm log

		PCA								t-SNE								UMAP							
		sG	H	L	Se	R	Sr	Si	A	sG	H	L	Se	R	Sr	Si	A	sG	H	L	Se	R	Sr	Si	A
ARI	CT	0.83	0.61	0.62	0.82	0.84	0.84	0.85	0.62	0.77	0.77	0.75	0.79	0.80	0.80	0.79	0.81	0.78	0.79	0.83	0.78	0.86	0.81	0.78	0.82
	1-B	1.00	1.00	1.00	1.00	1.00	1.00	1.00	1.00	1.00	1.00	1.00	1.00	1.00	1.00	1.00	1.00	1.00	1.00	1.00	1.00	1.00	1.00	1.00	1.00
	F1	0.76	0.76	0.77	0.90	0.91	0.91	**0.92**	0.77	0.87	0.87	0.86	0.88	0.89	0.89	0.88	**0.90**	0.87	0.88	0.90	0.88	**0.93**	0.89	0.88	0.90
ASW	CT	0.35	0.31	0.47	0.33	0.36	0.16	0.08	0.47	0.61	0.65	0.62	0.65	0.69	0.60	0.45	0.66	0.58	0.62	0.58	0.64	0.62	0.62	0.45	0.61
	1-B	1.00	1.00	1.00	1.00	0.97	0.98	0.99	1.00	1.00	1.00	1.00	1.00	1.00	1.00	0.99	1.00	1.00	1.00	1.00	1.00	1.00	1.00	0.99	1.00
	F1	0.52	0.48	**0.64**	0.50	0.53	0.16	0.15	**0.64**	0.76	0.79	0.77	0.79	**0.82**	0.75	0.62	0.80	0.73	0.76	0.74	**0.78**	0.76	0.76	0.62	0.76
LISI	CT	1.00	1.00	1.00	1.00	1.00	1.03	1.04	1.00	1.00	1.00	1.00	1.00	1.00	1.00	1.00	1.00	1.00	1.00	1.01	1.00	1.00	1.00	1.00	1.00
	B	1.92	1.89	1.94	1.75	1.34	1.77	1.77	1.90	1.94	1.93	1.97	1.85	1.60	1.80	1.75	1.95	1.93	1.92	1.97	1.87	1.86	1.76	1.72	1.93
	F1	0.85	0.84	**0.87**	0.77	0.48	0.78	0.75	0.86	0.86	0.86	**0.88**	0.82	0.68	0.80	0.77	0.87	0.86	0.86	**0.87**	0.83	0.83	0.78	0.76	0.86

(c) Dataset 1

		PCA							t-SNE							UMAP						
		sG	H	Se	R	Sr	Si	A	sG	H	Se	R	Sr	Si	A	sG	H	Se	R	Sr	Si	A
ARI	CT	0.79	0.70	0.70	0.37	0.74	0.74	0	0.76	0.73	0.70	0.76	0.87	0.80	0.83	0.69	0.68	0.72	0.72	0.83	0.82	0.74
	1-B	1.00	1.00	1.00	1.00	1.00	1.00	1.00	1.00	1.00	1.00	1.00	1.00	1.00	1.00	1.00	1.00	1.00	1.00	1.00	1.00	1.00
	F1	**0.88**	0.84	0.83	0.54	0.85	0.85	0	0.86	0.82	0.84	0.87	**0.93**	0.89	0.91	0.81	0.81	0.84	0.84	**0.91**	0.90	0.85
ASW	CT	0.56	0.28	0.27	0.27	0.21	0.10	-0.22	0.49	0.44	0.43	0.52	0.61	0.51	0.62	0.41	0.44	0.42	0.46	0.55	0.53	0.48
	1-B	0.98	0.99	0.99	0.98	0.96	0.96	1.00	0.98	0.99	1.00	0.97	0.95	0.96	0.95	0.97	0.98	1.00	0.97	0.95	0.97	0.95
	F1	**0.71**	0.44	0.43	0.42	0.35	0.17	-0.55	0.65	0.61	0.60	0.68	**0.75**	0.66	**0.75**	0.57	0.61	0.59	0.62	**0.70**	0.68	0.63
LISI	CT	1.00	1.00	1.00	1.00	1.00	1.09	1.00	1.01	1.01	1.01	1.00	1.00	1.00	1.01	1.02	1.01	1.01	1.02	1.00	1.00	1.01
	B	1.22	1.38	1.19	1.10	1.25	1.25	1.44	1.26	1.33	1.23	1.05	1.10	1.14	1.48	1.28	1.24	1.24	1.07	1.07	1.14	1.42
	F1	0.34	0.52	0.31	0.18	0.38	0.38	**0.57**	0.40	0.47	0.36	0.10	0.18	0.24	**0.60**	0.41	0.37	0.37	0.13	0.14	0.24	**0.55**

(d) Dataset 1 norm log

		PCA								t-SNE								UMAP							
		Sc	H	L	Se	R	Sr	Si	A	Sc	H	L	Se	R	Sr	Si	A	Sc	H	L	Se	R	Sr	Si	A
ARI	CT	0.74	0.71	0.62	0.72	0.85	0.83	0.72	0.71	0.82	0.78	0.80	0.70	0.66	0.78	0.51	0.81	0.83	0.78	0.61	0.69	0.78	0.70	0.64	0.81
	1-B	1.00	1.00	1.00	1.00	1.00	1.00	1.00	1.00	1.00	1.00	1.00	1.00	1.00	1.00	1.00	1.00	1.00	1.00	1.00	1.00	1.00	1.00	1.00	1.00
	F1	0.85	0.83	0.76	0.84	**0.92**	0.91	0.84	0.83	**0.90**	0.88	0.89	0.83	0.79	0.88	0.68	0.89	**0.91**	0.88	0.76	0.82	0.88	0.83	0.78	0.90
ASW	CT	0.36	0.27	0.44	0.21	0.30	0.14	0.03	0.35	0.64	0.58	0.63	0.47	0.34	0.54	0.25	0.57	0.66	0.58	0.51	0.47	0.48	0.51	0.29	0.54
	1-B	0.97	0.98	0.92	0.99	0.94	0.97	0.99	0.99	0.95	0.96	0.87	1.00	0.97	0.97	0.98	0.98	0.95	0.98	0.92	1.00	0.99	0.97	0.97	0.98
	F1	0.52	0.42	**0.59**	0.35	0.46	0.25	0.05	0.51	**0.76**	0.72	0.73	0.64	0.50	0.69	0.40	0.72	**0.79**	0.73	0.65	0.64	0.65	0.67	0.45	0.70
LISI	CT	1.00	1.00	1.00	1.00	1.00	1.04	1.66	1.00	1.00	1.00	1.00	1.00	1.00	1.04	1.15	1.00	1.00	1.00	1.01	1.00	1.00	1.00	1.01	1.00
	B	1.45	1.49	1.49	1.25	1.03	1.42	1.57	1.52	1.49	1.60	1.33	1.42	1.36	1.30	1.71	1.66	1.52	1.58	1.39	1.49	1.53	1.39	1.26	1.58
	F1	0.58	0.61	0.61	0.39	0.05	0.55	0.62	**0.63**	0.61	0.69	0.47	0.56	0.50	0.44	**0.74**	0.73	0.63	**0.67**	0.53	0.61	0.64	0.52	0.40	**0.67**

(e) Dataset 2 norm log

		PCA								t-SNE								UMAP							
		Sc	H	L	Se	R	Sr	Si	A	Sc	H	L	Se	R	Sr	Si	A	Sc	H	L	Se	R	Sr	Si	A
ARI	CT	0.96	0.95	0.86	0.95	0.95	0.95	0.95	0.95	0.96	0.95	0.83	0.93	0.94	0.93	0.92	0.95	0.96	0.95	0.86	0.94	0.93	0.95	0.95	0.95
	1-B	0.99	1.00	1.00	0.99	0.99	0.97	0.95	0.99	0.99	0.98	1.00	0.99	0.98	0.96	0.94	0.99	0.99	0.99	1.00	0.99	0.98	0.95	0.94	0.99
	F1	**0.98**	0.97	0.92	0.97	0.97	0.95	0.93	0.97	**0.97**	**0.97**	0.90	0.95	0.96	0.94	0.93	**0.97**	**0.98**	0.97	0.92	0.96	0.95	0.95	0.94	0.97
ASW	CT	0.25	0.17	0.35	0.23	0.34	0.15	0.02	0.23	0.34	0.29	0.27	0.47	0.48	0.30	0.27	0.25	0.55	0.45	0.45	0.54	0.52	0.51	0.39	0.46
	1-B	1.14	1.11	1.14	1.14	1.12	1.09	1.13	1.11	1.10	1.08	1.08	1.06	1.06	1.07	1.12	1.04	1.06	1.04	1.06	1.03	1.06	1.07	1.14	1.07
	F1	0.23	0.29	**0.53**	0.38	0.52	0.26	0.03	0.38	0.52	0.46	0.43	0.65	**0.66**	0.47	0.43	0.40	**0.72**	0.63	0.63	0.71	0.70	0.69	0.58	0.65
LISI	CT	1.00	1.00	1.00	1.00	1.00	1.00	1.02	1.00	1.00	1.00	1.01	1.00	1.00	1.00	1.00	1.00	1.00	1.00	1.01	1.00	1.00	1.00	1.00	1.00
	B	1.53	1.59	2.38	1.75	1.73	1.19	1.28	1.59	1.42	1.35	2.40	2.16	1.84	1.05	1.05	1.54	1.49	1.50	2.36	1.61	1.59	1.08	1.04	1.68
	F1	0.23	0.26	**0.51**	0.31	0.31	0.09	0.13	0.26	0.19	0.16	**0.51**	0.44	0.34	0.02	0.02	0.24	0.22	0.22	**0.50**	0.26	0.26	0.09	0.02	0.29

The metrics are evaluated on the PCA, t-SNE, and UMAP embeddings of the full corrected data for scGen (sG), Harmony (H), LIGER (L), Seurat (Se), ResPAN (R), scVI (r) (Sr), scVI (i) (Si) and AIF dyn (A). Each metric is computed for the cell type purity (CT), the batch mixing (B), and combining both criteria (F1).

To better understand the embedding algorithms' bias, we selected cases in which the clustering results outputted by K-Means were drastically different across the different embeddings' subspaces and illustrated them in Figs C and D. In Fig C, we observe that the PCA algorithm placed cells from different cell types in the same subspace's area based on their second principal component's value. This is not observed in the t-SNE embeddings, showing that using PCA, in this case, would wrongfully deteriorate the results. In Fig D, the UMAP algorithm placed a group of different cell types, probably corresponding to one of the artifact clusters induced by the pre-processing normalization step, at the top right of the plot with a strong expression of the two first UMAP's components. This led K-Means to cluster CD141+ and pDC cells together and call the artefact cluster as if it were a particular cell population, which is not observed in the t-SNE subspace as the artefact cluster remains close to the pDC cluster.

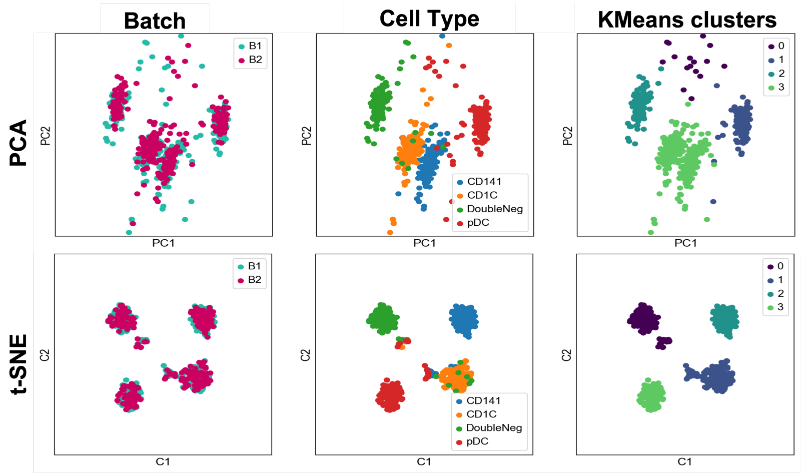

Figure C. Illustration of the PCA's bias in scGen's K-Means clustering. The results correspond to the best clustering on randomly sampled 80% of scGen's corrected data trained on Dataset 0 norm log.

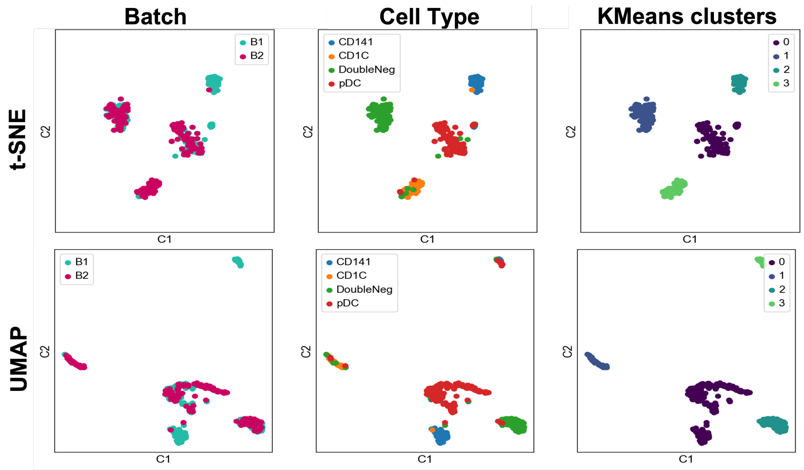

Figure D. Illustration of the UMAP's bias in LIGER's K-Means clustering. The results correspond to the best clustering on randomly sampled 80% of LIGER's corrected data trained on Dataset 1 norm log.

3 K-Means' failures

3.1 Small datasets

To illustrate specifically the bias induced by the K-Means algorithm, we represented the most striking example in Fig E, where K-Means failed to accurately cluster the cells whereas Louvain successfully retrieves the true underlying clusters in AIF dyn's corrected data trained on Dataset 1 with $\alpha = 0.01, \rho = 0.4, \beta = 0.05, \gamma = 0.4, \delta = 0.2$, $\mu = 0$, and a batch size of 32, a learning rate of 0.01, $\beta_1 = 0.9$ and $\beta_2 = 0.95$.

We observe in Fig E that K-Means struggles to separate the CD1c+ cluster from the CD141+ one in the t-SNE, even though the two cell types visually belong to distinct clusters. As K-Means search for a pre-determined number of clusters, it results in a division of the pDC cluster, which is visually homogeneous and should not be divided. We note that the clustering is satisfying using Louvain, confirming that the loss of information is not due to the batch-effect correction but solely to the clustering algorithm's performance. Thus, K-Means failed to capture most biological information in the t-SNE subspace, deteriorating any downstream clustering metric and biasing the results.

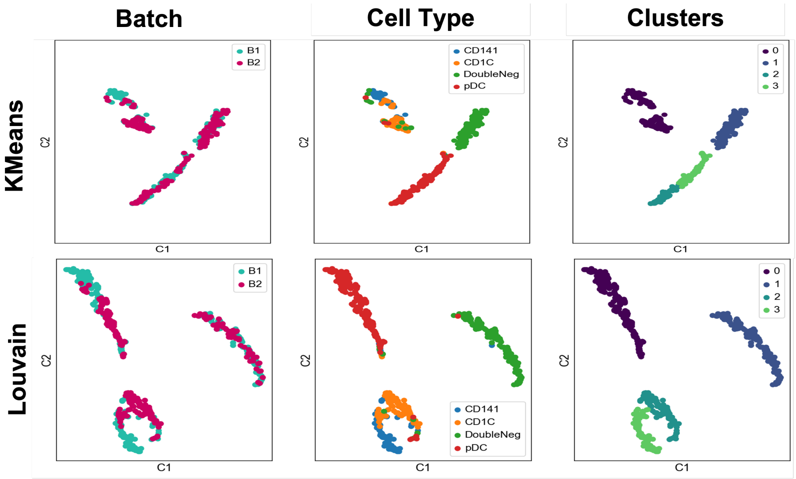

Figure E. Illustration of a K-Means' failure in the t-SNE subspace. The results correspond to the best clustering on randomly sampled 80% of AIF dyn's corrected data, trained on Dataset 1, where Louvain successfully clusters the cells.

3.2 Large dataset

In this section, we illustrated in Fig F a K-Means' failure in the t-SNE subspace for scGen's corrected data trained on Dataset 2 norm log, as it is supposed to have the best-separated clusters.

We observe in Fig F that K-Means is unable to detect the clusters having few cells and merge them into one big cluster (e.g., endothelial, macrophage, and mast cluster). Furthermore, as the number of clusters is pre-determined, this results in multiple splits of the clusters corresponding to highly represented cell types (e.g., alpha, beta, etc.), yielding meager results regarding ARI-related metrics. In comparison, Louvain can retrieve small clusters (e.g., mesenchymal cluster or separate macrophage and mast cells from the endothelial cluster) while keeping cells from large clusters together (e.g., alpha, beta, and acinar cells). This shows that using K-Means would unfairly penalize the models and make the comparison less relevant.

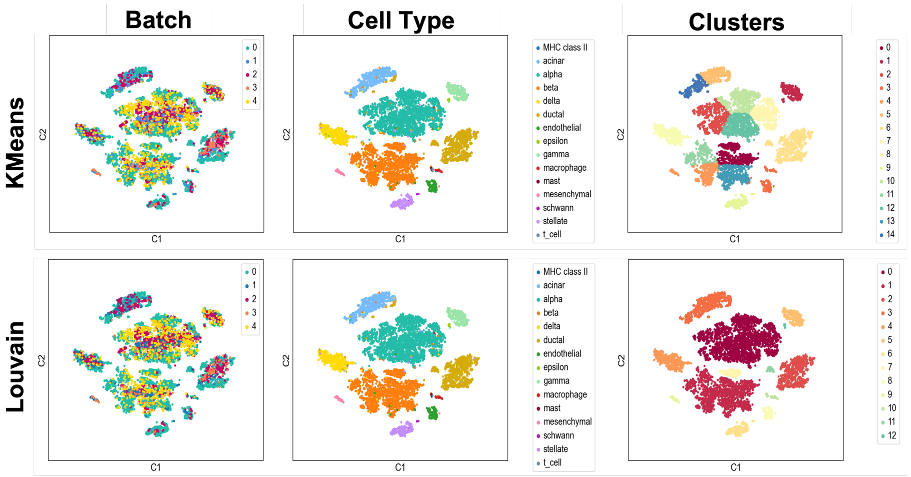

Figure F. Illustration of a K-Means' failure in the t-SNE subspace. The results correspond to the best clustering on randomly sampled 80% of scGen's corrected data trained on Dataset 2 norm log and are compared to Louvain clustering results with a dichotomy search guided by the cell type ARI.

4 Louvain's bias

4.1 Louvain's hyperparameters

Although the Louvain algorithm improved the fairness of models' comparison for the Dataset 2 norm log, its performance is sensitive to the hyperparameters and the initial graph used, which we will try to highlight in this section. In Fig G, we represented t-SNE visualizations corresponding to the best F1 ARI score over the 20 experiments for different configurations of Louvain's hyperparameters, running the evaluation pipeline on the AIF dyn's corrected data for Dataset 2 norm log, on either the training set or the training and test sets, with a dichotomy search guided by the cell type ARI. We explored different values for the aggregation's and optimization's tolerance (tol), the number of neighbors (N) to construct the initial kNN graph, and using weighted edges in the initial graph (referred to as w-kNN in the figure). We varied the maximum number of iterations, but it did not change the results, indicating that 20 iterations are enough for the algorithm to converge.

Although the tolerance parameter is the stopping criterion in Louvain's aggregation and optimization steps, it does not impact the clustering either on the training or complete dataset, highlighting the clustering pipeline's robustness to this hyperparameter. When performing the dichotomy search on the number of clusters, decreasing the tolerance led to more fine-grained partitions, better capturing the rare cell types. However, it also sub-divided other clusters by calling small isolated clusters. Thus, we kept the value of $tol = 1e-4$ to incite the algorithm to capture rare populations, which yielded great results with the dichotomy search on the number of clusters.

The number of neighbors in the kNN graph plays a crucial role as Louvain relies on the corresponding adjacency matrix. We observe in Fig G that it controls the partition's resolution. Increasing this parameter yields a more coarsened version of the clustering, leading to the merger of the two beta clusters in the entire dataset. However, the macrophage cells are now clustered with the delta cells, and more alpha cells are subdivided. On the training set, this number of neighbors does not change the clustering results, indicating some robustness to this parameter. Thus, we retained the value of 20 neighbors.

Although kNN is a good baseline for initializing the graph, it relies on a fixed number of neighbors for each node, regardless of the distance. To fully benefit from all the information available, we investigated a weighted kNN graph whose edges are inversely proportional to the Euclidean distance. It results in a total separation of the macrophage and delta cells on both the training and full datasets, but also of the stellate and endothelial cells in the complete set, thus improving the clustering results. One might consider using the weighted kNN graph for a fairer comparison of

the models. Nevertheless, the clustering results with kNN already provide a reasonable basis for comparison purposes.

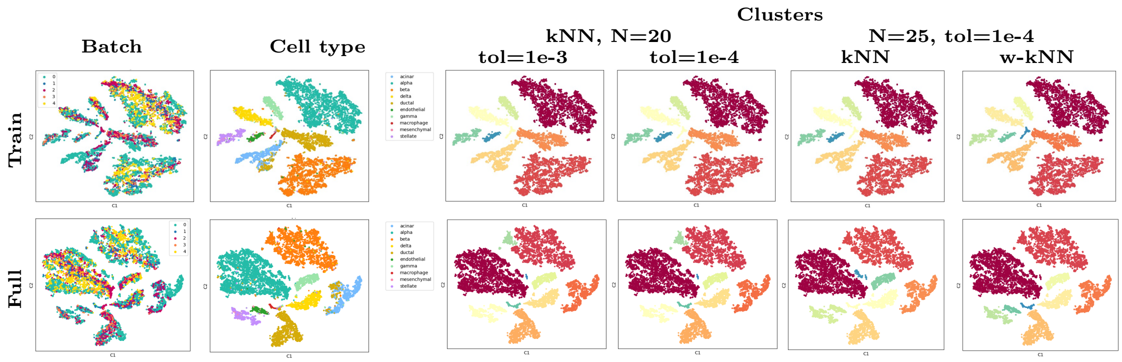

Figure G. Illustration of Louvain's sensitivity to its hyperparameters. The results correspond to the best clustering on the t-SNE subspace of AIF dyn's training or full (training and test sets) corrected data on Dataset 2 norm log: aggregation's and optimization's tolerance (tol), number of neighbors (N) in the initial kNN graph, using weighted edges in the kNN graph (w-kNN).

4.2 Comparison with Leiden

To further assess the extent of Louvain's bias, we explored a more sophisticated clustering algorithm called Leiden [1], a refinement of Louvain. We compared the results of each clustering algorithm on the model's corrected data on the human pancreas dataset (Dataset 2 norm log) in Fig H, whether subsampling 80% of the data or using the entire dataset. As expected, only the ARI metrics are affected since the others are independent of the clustering algorithm's outputs. Although Leiden yields more stable and higher results on the subsampled dataset, it is not verified on the entire dataset, where no clear trend can be established. Thus, Louvain's bias is relatively small and would not be alleviated by switching to Leiden.

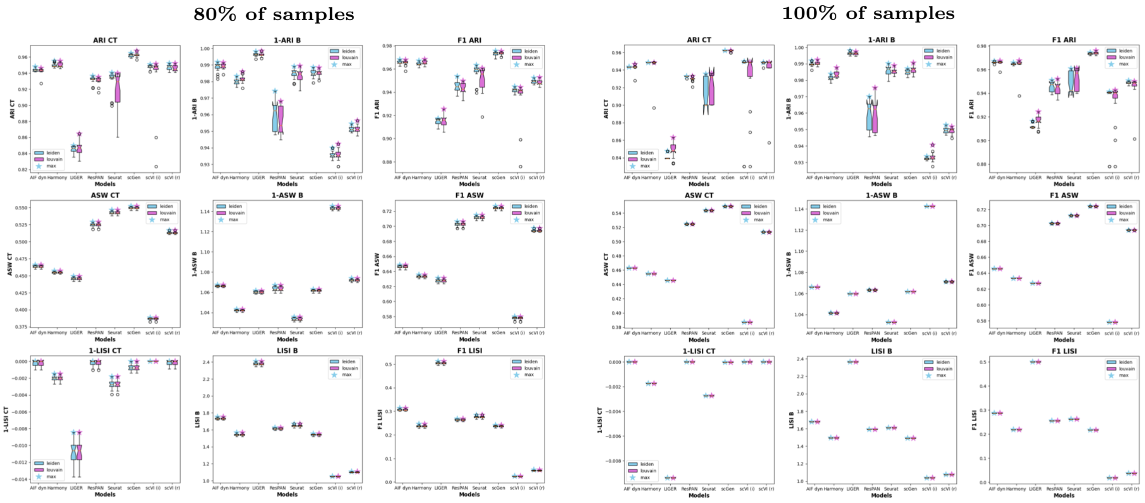

Figure H. Comparison of Louvain and Leiden clustering metrics on the corrected Dataset 2 norm log. The metrics are calculated 20 times on randomly sampled 80% or 100% of the corrected data's UMAP embeddings for dataset 2's log-normalized counts. Each metric is computed for the cell type purity (CT), the batch mixing (B), and combining both criteria (F1).

References

1. Traag VA, Waltman L, van Eck NJ. From Louvain to Leiden: guaranteeing well-connected communities. Scientific Reports. 2019;9(1):5233. doi:10.1038/s41598-019-41695-z.
