# Supplementary material for: A novel batch-effect correction method for scRNA-seq data based on Adversarial Information Factorization
Source: PLoS Comput Biol. 2024 Feb 22;20(2):e1011880. doi: 10.1371/journal.pcbi.1011880 (PMC10914288; doi:10.1371/journal.pcbi.1011880)
Supplement: S6 Appendix — This appendix presents the clustering results before the DE analysis and delves into the DE results. (PDF) [file pcbi.1011880.s006.pdf]

S6 Appendix: Differential Expression analysis

Lily Monnier[1], Paul-Henry Cournède[1,*]

1. MICS Laboratory, CentraleSupélec, Paris-Saclay University, Gif-sur-Yvette, France.

* corresponding author: paul-henry.cournede@centralesupelec.fr

1 Clustering results

Before running the Differential Expression (DE) analysis, we performed K-Means in the t-SNE subspace of the original (Raw (u)) and each model's corrected data. We used Louvain with 50 neighbors for kNN to fairly evaluate the DEGs detection performance when K-Means failed to cluster the data due to the clusters' shape (see Table A). The corresponding metrics are reported in Table B. We also incorporated the results of the supervised DE analysis, i.e. using the true cell type labels, for the original data (Raw (s)). In opposition to what is presented in [1], the batch effects are not high enough to confound the supervised DEG detection, leading to high F1 scores. Thus, the supervised uncorrected DEGs' results would constitute a good sanity check for the batch-effects correction methods' preservation of the biological signal.

We observe that all batch-effect-corrected data yield perfect clustering in terms of ARI, meaning that the DE analysis results solely reflect the models' ability to preserve the cell types' gene expression and are not impacted by the clustering algorithm's performance. The low performance on the original data is due to the mixing of two cell types, as observed in the corresponding t-SNE visualization in Fig A. We note that ResPAN yields inferior batch mixing results, outlined by lower batch LISI scores.

Moreover, ResPAN's batch-effect correction focuses only on the top 2,000 HVGs detected in either of the two batches. We completed the corrected data with the uncorrected original data to integrate ResPAN into the DE analysis comparison. The additional genes still suffer from batch effects and lead to a higher separation of the cell types corresponding to their batch in the visualization but do not impact the clustering labels as the clustering algorithm groups them together (Fig B). Note that more than half of the genes tested are left untouched by ResPAN and correspond to the raw supervised method, which arbitrarily advantages this model since the batch effects do not confound the DEG analysis.

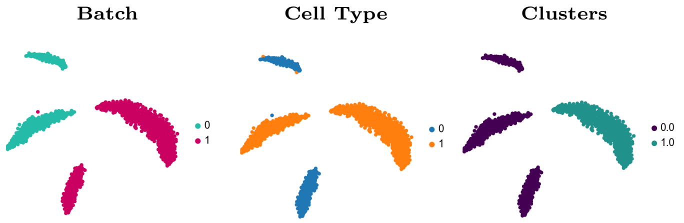

Figure A. Unsupervised clustering on raw data. t-SNE visualization of the uncorrected Dataset 3's raw counts. The cells are colored by batch (left), cell type (middle) or K-Means clusters (right).

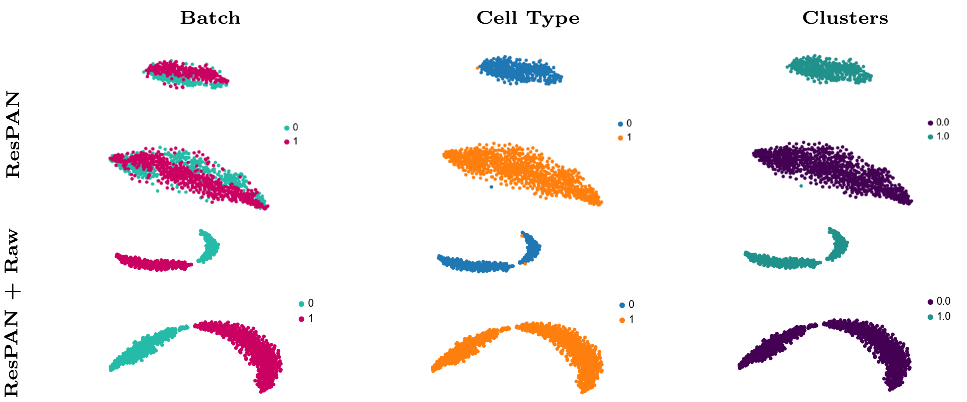

Figure B. Unsupervised clustering on ResPAN corrected data. t-SNE visualization of ResPAN's corrected data (ResPAN) before and after having completed it with the uncorrected data (ResPAN + Raw) on Dataset 3's raw counts. The cells are colored by batch (left), cell type (middle) or K-Means clusters (right).

Table A. Clustering algorithm for each model and dataset.

	Dataset 3		Dataset 3 ($n_1 = 200$)		Dataset 3 ($n_1 = 100$)		Dataset 4	
	KMeans	Louvain	KMeans	Louvain	KMeans	Louvain	KMeans	Louvain
Raw (u)	✓		✓		✓		✓	
ResPAN	✓		✓			✓	✓	
scGen		✓		✓		✓	✓	
Seurat	✓		✓		✓		✓	
AIF dyn	✓			✓		✓		✓

Table B. Comparison of the methods' clustering results on the full dataset.

Dataset 3

	ARI			ASW			LISI		
	CT	1-B	F1	CT	1-B	F1	CT	B	F1
Raw (u)	0.25	0.61	0.35	0.53	0.72	0.61	1.00	1.00	0
ResPAN	0.99	1.00	**1.00**	0.65	1.00	0.79	1.00	1.25	0.36
scGen	0.99	1.00	**1.00**	0.48	1.00	0.65	1.00	1.69	0.68
Seurat	1.00	1.00	**1.00**	0.79	1.00	**0.88**	1.00	1.80	0.73
AIF dyn	0.99	1.00	**1.00**	0.47	1.00	0.64	1.00	1.84	**0.74**

Dataset 3 ($n_1 = 200$)

	ARI			ASW			LISI		
	CT	1-B	F1	CT	1-B	F1	CT	B	F1
Raw (u)	0.09	0.52	0.15	0.52	0.55	0.53	1.00	1.00	0
ResPAN	0.99	0.99	**0.99**	0.64	0.95	0.77	1.00	1.10	0.17
scGen	0.99	1.00	**0.99**	0.32	1.00	0.49	1.00	1.68	0.67
Seurat	1.00	0.99	**0.99**	0.78	0.99	**0.87**	1.00	1.83	0.74
AIF dyn	0.99	0.99	**0.99**	0.49	0.99	0.66	1.00	1.87	**0.75**

Dataset 3 ($n_1 = 100$)

	ARI			ASW			LISI		
	CT	1-B	F1	CT	1-B	F1	CT	B	F1
Raw (u)	0.09	0.27	0.13	0.44	0.47	0.46	1.00	1.00	0
ResPAN	0.99	0.99	0.99	0.61	0.93	0.74	1.00	1.06	0.12
scGen	0.99	1.00	**1.00**	0.36	0.99	0.53	1.00	1.55	0.60
Seurat	0.99	1.01	0.99	0.68	0.99	**0.81**	1.00	1.83	**0.74**
AIF dyn	0.99	1.00	0.99	0.40	1.00	0.57	1.00	1.83	**0.74**

Dataset 4

	ARI			ASW			LISI		
	CT	1-B	F1	CT	1-B	F1	CT	B	F1
Raw (u)	0.27	0.64	0.38	0.52	0.70	0.59	1.00	1.00	0
ResPAN	1.00	1.00	**1.00**	0.65	1.00	0.78	1.00	1.17	0.27
scGen	1.00	1.00	**1.00**	0.50	1.00	0.67	1.00	1.70	0.68
Seurat	0.99	1.00	**1.00**	0.74	1.00	**0.85**	1.00	1.83	**0.74**
AIF dyn	1.00	1.00	**1.00**	0.43	1.00	0.60	1.00	1.84	**0.74**

The metrics are evaluated on the t-SNE embeddings of Dataset 3's corrected raw counts. Each metric is computed for the cell type purity (CT), the batch mixing (B), and combining both criteria (F1).

2 Differential Expression results

2.1 Estimation of the log-fold-change

To further evaluate the models' DE performance, we investigated the faithfulness of the log-fold-change estimation on both versions of the counts, considering all the genes or the HVGs only. Fig C depicts the correlation between the inferred and the actual log-fold-change of differentially expressed genes (DEGs) for each model's corrected data and the corresponding MSE.

AIF dyn outperforms all the other models on the log-normalized counts. The higher quality of estimating the predicted log-fold change can be assessed with the better alignment of the points on the bisector, yielding a lower MSE on both the HVGs and all genes. Seurat batch-effect correction attenuates the biological difference between the two cell types for some genes, resulting in more undetected DEGs, especially for the HVGs. Although scGen, ResPAN, and AIF dyn yield more false DEGs, they correspond to small estimated log-fold-change, which would be easily filtered out with a small log-fold-change threshold. ResPAN's results on all genes resemble the Raw (s) model, as more than half of the genes are left uncorrected, which makes it difficult to assess the quality of the correction truly. On the HVGs, the difference with Raw (s) is more significant as most of the genes are corrected, showing the actual deterioration of the biological signal induced by ResPAN's batch-effect correction compared to the uncorrected data.

On the raw counts, all models yield satisfying estimates of the log-fold change on the HVGs. Still, none surpasses the Raw (s) model since the batch effects do not confound the DE analysis on the HVGs' raw counts (perfect alignment of the points on the bisector). Seurat gives the best results among the corrected data. AIF dyn tends to slightly overestimate the log-fold-change, especially for highly differentiated genes, resulting in a marginally higher MSE than ResPAN but still lower than scGen. On all genes, Seurat and AIF dyn slightly outperform the Raw (s) model, and AIF dyn's log-fold-change estimates are the most aligned on the bisector. ResPAN and scGen yield poorer estimation of the log-fold-change due to high falsely detected DEGs and high undetected DEGs for ResPAN only.

To conclude, AIF dyn provides the corrected data with the best estimation of the log-fold change on the log-normalized counts while yielding top-ranked results on the raw counts.

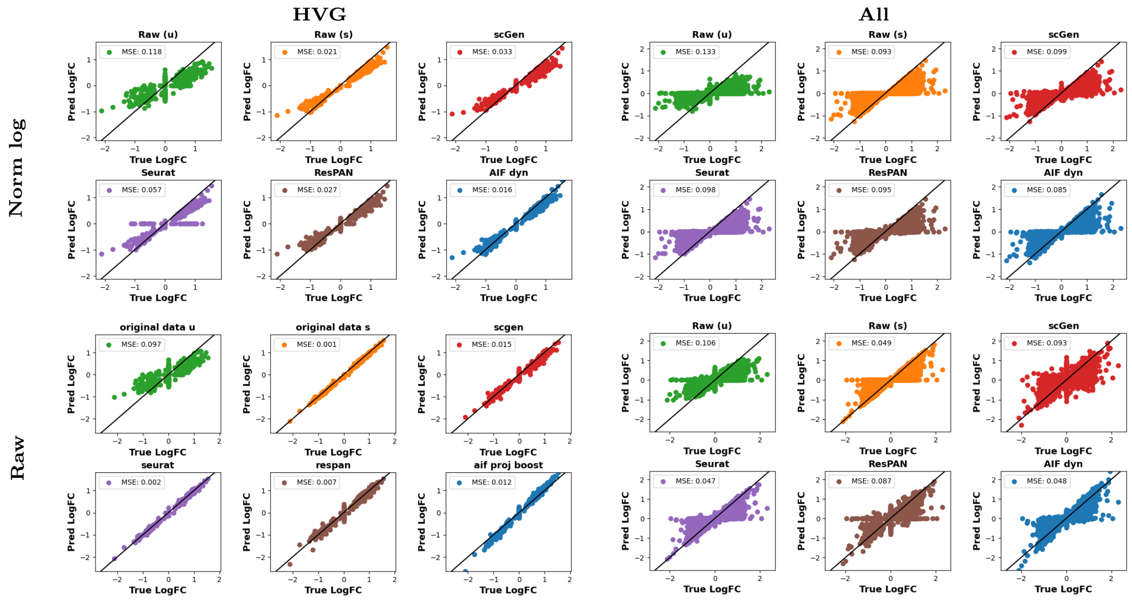

Figure C. Comparison of the log-fold-change estimates. Correlation between the inferred and the true log-fold-change of DEGs for each model's corrected raw and log-normalized counts on Dataset 3.

2.2 Evaluation on ResPAN's genes subset

In this section, we focus on the DE results on ResPAN's genes subset only to evaluate the models more fairly. Indeed, since ResPAN does not correct all genes, its intrinsic performance cannot be assessed as such when including the other genes and greatly depends on the results of the uncorrected

data. As the Raw (s) model yields great DE results, it unfairly advantages ResPAN, especially on the raw counts. Thus, to assess solely ResPAN batch-effect correction, we computed the metrics on the genes corrected by ResPAN only and compared the models' log-fold-change estimates on this genes subset (Fig D).

First, the difference with the Raw (s) model is more significant when focusing on those genes only since they are no longer diluted by the Raw (s) results on the other genes. It underlines the unfairness of comparison when including all the genes for ResPAN and highlights the biological deterioration induced by ResPAN's batch-effect correction.

On this genes' subset, AIF dyn outmatches all the other models on both the log-normalized and raw counts. The difference in performance is more significant than on the HVGs or on all genes, which emphasizes its superiority.

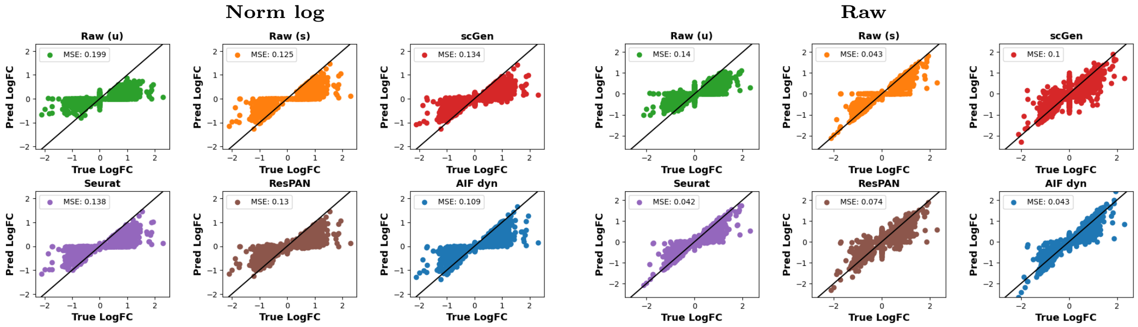

Figure D. Comparison of the log-fold-change estimates on ResPAN's genes subset. Correlation between the inferred and the true log-fold-change of DEGs for each model's corrected raw and log-normalized counts on Dataset 3 restricted to the subset of genes outputted by ResPAN.

References

1. Tran HTN, Ang KS, Chevrier M, Zhang X, Lee NYS, Goh M, et al. A benchmark of batch-effect correction methods for single-cell RNA sequencing data. Genome Biology. 2020;21(1):12. doi:10.1186/s13059-019-1850-9.
